# Designing Diaryl Sulfide Electronic Properties: A Comprehensive DFT Guide to HOMO–LUMO Gaps and Reactivity Descriptors

**DOI:** 10.1021/acsomega.5c11432

**Published:** 2026-05-01

**Authors:** Ramon S. da Silva

**Affiliations:** Departamento de Física, 28113 Universidade Federal de Juiz de Fora, Juiz de Fora, 36036-330, Minas Gerais, Brazil

## Abstract

Diaryl sulfides, characterized by a sulfur atom bonded to two aryl groups, exhibit distinctive electronic properties arising from the interplay between the delocalized π-system of the aromatics and the lone pairs on sulfur. We report the first comprehensive computational study of 12 diaryl sulfide (DS12) derivatives using nine density functional theory (DFT) functionals. Their geometric parameters (bond distances, angles, and torsion angles), dipole moments, vertical transition energies, and thermochemical properties (ionization potentials and electron affinities) were computed. In the absence of experimental data, the DFT results were benchmarked against high-level DLPNO–CCSD­(T)/cc-pVTZ calculations. The analysis reveals that molecule **8** is the most stable (HOMO–LUMO gap = 4.358 eV), whereas molecule **1** exhibits the highest electrophilicity (6.931 eV). For dipole moment calculations, B1LYP emerges as the most accurate functional (MAE = 0.246 D), closely followed by B3LYP (MAE = 0.262 D). In contrast, the TPSSh (MAE ≈ 0.20 eV) and B3PW91 (MAE ≈ 0.17 eV) functionals are recommended for exploratory studies requiring reliable vertical transition energies.

## Graphical abstract

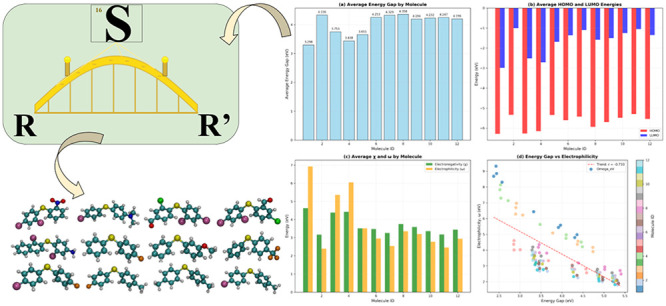

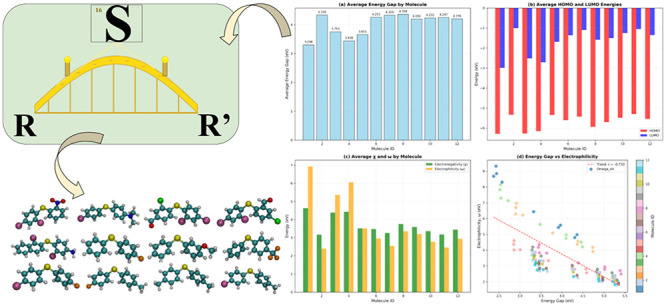

## 1 Introduction

Sulfur is the second lightest element of group 16 of the periodic table (group VI of the main *p*-block elements), known as the *chalcogens*, which also includes oxygen, selenium, tellurium, and polonium. This nonmetal, with atomic number 16 and the electronic configuration [Ne]­3s^2^3p^4^, possesses a filled 3s orbital and four electrons in the 3p orbitals. This configuration leads to a tendency to gain two electrons to achieve a noble gas configuration, underpinning its diverse bonding capabilities and reactivity. Despite its common association with toxic compounds such as hydrogen sulfide (H_2_S) and sulfur dioxide (SO_2_), elemental sulfur itself is renowned for its low toxicity and is essential for biological processes.^1,2^

The versatile chemistry arising from this electronic structure makes sulfur-containing organic compounds a cornerstone of modern chemical science. Their applications span a vast range of fields, including pharmaceuticals,^3,4^ catalysis,^5^ materials science,^6–8^ and spectroscopy.^9–11^ The diverse biological activities of these compounds, such as HIV protease inhibition and anti-inflammatory effects, make them prominent targets for drug discovery.^12^ Among them, diaryl sulfides serve as fundamental structural motifs and valuable synthetic intermediates.

Despite the characteristically pyramidal geometry of the sp^3^-hybridized sulfur center, which introduces torsional flexibility, sulfur acts as an effective “electronic bridge” in diaryl sulfides. This nonplanarity does not hinder strong electronic communication; rather, the polarizable sulfur lone pairs conjugate with the aromatic π-systems through hyperconjugation, enabling a tunable interplay of electron donation and delocalization that governs the molecule’s optoelectronic properties.

Diaryl sulfides represent an important class of compounds with significant applications in diverse fields such as materials science,^13^ corrosion inhibition,^14^ and medicinal chemistry.^15,16^ Their relevance in pharmacology is exemplified by pharmaceuticals like the antidepressant LuAA21004 ^17^ and Axitinib, a medication used to treat advanced renal cell carcinoma (RCC).^18^ In addition, chlorbenside is a key compound in this class and is widely used as an industrial pesticide.^19,20^

Experimentally, several synthetic methodologies have been developed for the preparation of diaryl sulfides. Metal-catalyzed cross-coupling represents a particularly powerful route to diaryl sulfides.^21–27^ Transition metals can efficiently activate organic compounds, enabling the catalytic formation of new bonds. A representative example is the work of Zhang and co-workers,^28^ who demonstrated the synthesis of aryl sulfides via a copper-catalyzed reaction between *N*-arylthiosuccinimides and arylaluminum reagents at temperatures close to 60 °C. Alternatively, Liang et al.^29^ reported a Pd-catalyzed, Ni-mediated strategy for the synthesis of aryl sulfides, which was demonstrated to be highly efficient.

Xiao et al.^30^ reported the use of l-cysteine, an α-amino acid, as a sulfur source in a transition-metal-catalyzed C–S cross-coupling for the synthesis of diaryl sulfides. Separately, Liu et al.^31^ synthesized several diaryl sulfides and characterized them using NMR spectroscopy, recorded on a General Electric QE300 instrument, alongside mass spectral analyses employing various techniques including desorption chemical ionization (DCI).

Despite the growing interest in diaryl sulfides, a systematic investigation of their geometrical parameters and key molecular properties such as their chemical bonding, energy gap (HOMO–LUMO), electron affinity (EA), and ionization potentials (IP) is lacking. For example, the HOMO–LUMO gap is a critical parameter governing electronic behavior. In aromatic systems, increased π-conjugation typically reduces this gap, resulting in a bathochromic shift of optical absorption; this principle is fundamental for designing organic semiconductors and dyes.^32^ Moreover, this energy gap serves as a key indicator of a molecule’s kinetic stability and chemical reactivity.

This study reports the first comprehensive investigation into the geometric, electronic, and thermochemical properties of a systematically designed series of 12 diaryl sulfide derivatives (DS12), employing density functional theory (DFT) calculations. The molecular set was strategically constructed to span a diverse range of substituent effects, including strong electron-withdrawing groups (NO_2_, CF_3_, halogens), electron-donating groups (NH_2_, OCH_3_), and neutral moieties, as well as symmetric and asymmetric substitution patterns. By analyzing frontier molecular orbital energies and global reactivity descriptors such as electronegativity (χ), chemical hardness (η), and electrophilicity (ω), we elucidate clear structure–property relationships, providing essential insights for the targeted molecular design of functional diaryl sulfide-based systems.

## 2 Computational Details

All quantum chemical calculations were performed using the ORCA program package.^33,34^ A comprehensive benchmark study was conducted to identify the most suitable density functional theory method for describing the molecular properties of the selected diaryl sulfide derivatives. The investigation encompassed a broad range of exchange-correlation functionals spanning different rungs of Jacob’s Ladder, including generalized gradient approximation (GGA) (e.g., BP86,^35^ PBE,^36^ BLYP^37^), meta-GGA (TPSS^38^), global hybrid GGA (B3LYP,^39,40^ B1LYP,^37,39^ PBE0,^41^ B3PW91 ^42^), and global hybrid meta-GGA (TPSSh^43^). Additional calculations were performed to evaluate the performance of the GFN2-*x*TB semiempirical method^44^ in assessing its accuracy for optimized molecular geometries and electronic properties relative to established DFT approaches.

All DFT calculations employed the def2-TZVPP basis set^45^ and the corresponding auxiliary def2-TZVPP/C basis set. Geometry optimizations were performed for all species, followed by vibrational frequency analyses at the same level of theory to confirm the nature of the stationary points. We also assessed the effect of including Grimme’s D3 dispersion correction^46^ with Becke-Johnson damping^47^ on the optimized molecular structures.

A comparative assessment of each functional’s performance was carried out by evaluating the calculated dipole moments against high-level coupled-cluster reference values obtained using the domain-based local pair natural orbital CCSD­(T) [DLPNO–CCSD­(T1)] method.^48,49^ In this method, the iterative computation of triples amplitudes provides accuracy approaching that of canonical (T) energies.^50^ It is well-known that the CCSD­(T) method exhibit computational scaling of O­(N^7^), where N is a measure of the system size. Therefore, to avoid the high computational cost associated with DLPNO–CCSD­(T) geometry optimizations, a protocol similar to that of Shirazi and co-workers^51^ was employed throughout this study. Thus, single-point DLPNO–CCSD­(T) energy and property calculations were performed at the B3LYP/def2-TZVPP optimized geometries using the cc-pVTZ basis set^52^ with recommended TightPNO settings.^53^ Additionally, while DLPNO–CCSD­(T) closely approaches canonical CCSD­(T) accuracy, small deviations may remain for systems with pronounced multireference character.

Vertical excitation energies for the DS12 test set were calculated using time-dependent density functional theory (TDDFT)^54^ within the Tamm–Dancoff approximation (TDA),^55^ which is the default method for excited-state calculations in the ORCA program package. To assess the impact of this approximation on our results, we also performed selected calculations using the full TDDFT formalism (i.e., without the TDA) for comparative purposes. These TDDFT results were then benchmarked against high-level wave function-based calculations performed with the transformed equation of motion method, augmented by the domain-based local pair natural orbitals approach (STEOM-DLPNO–CCSD),^48,56^ a method capable of accurately treating excited-state energies through its inclusion of higher-order singles and doubles excitations. The advanced STEOM-DLPNO–CCSD method was employed with the cc-pVTZ basis set and TightPNO settings to ensure convergence.

Global reactivity descriptors, including electronegativity (χ), chemical hardness (η), chemical potential (μ), softness (*S*), and the electrophilicity index (ω), were computed for all DFT functionals. These descriptors were derived from the ionization energy (*I*) and electron affinity (*A*), approximated as *I* ≈−ϵ_HOMO_ and *A* ≈−ϵ_LUMO_, respectively. The remaining descriptors are defined as follows:^57^

χ=(I+A)/2 1

η=(I−A)/2 2

μ=−χ 3

$$\omega =\mu^{2}/\left(2\eta \right)$$ 4

S=1/(2η) 5

The HOMO–LUMO energy gap (*E*_HOMO_ – *E*_LUMO_) was analyzed as a key indicator of kinetic stability and chemical reactivity. It should be noted that this relationship, while useful for identifying trends within a series of related compounds, is a conceptual tool rather than a direct thermodynamic stability metric.

Alternatively to the Koopmans’ theorem, the ionization potential (IP_*N*_) and electron affinity (EA_*N*_) of a neutral *N*-electron system can be computed via energy differences.^58–60^ The IP_*N*_, corresponding to the energy required to remove an electron, is calculated as IP_*N*_ = *E*(*N* – 1) – *E*(*N*). Conversely, the EA_*N*_, which represents the energy released upon adding an electron (or the detachment energy from the anion), is given by EA_*N*_ = *E*(*N*) – *E*(*N* + 1), where *E*(*N*) denotes the total energy of the *N*-electron system.

This energy-difference approach generally yields more accurate IP and EA values than Koopmans’ theorem, as it explicitly accounts for electronic relaxation and structural reorganization in both the *N* – 1 and *N* + 1 electron systems. Standard calculations employed the def2-TZVPP basis set; however, to obtain more reliable electron affinities, we conducted benchmark calculations with the def2-TZVPPD basis set, including diffuse functions in the calculations.

The following section provides a detailed assessment of the functional performance through statistical parameters, which include the mean absolute error (MAE), root-mean-square error (RMSE), and standard deviation (SD). Detailed equations for these metrics are available in the Supporting Information (SI). Figure S1 in the Supporting Information schematically outlines the overall computational workflow for this DFT investigation. Furthermore, Figure S2 directly compares the computational costs, presenting the typical CPU times required for geometry optimizations of three diaryl sulfides across nine density functionals.

The data in Figure S2 reveal that pure functionals, particularly BLYP, consistently achieve the fastest calculations, whereas hybrid functionals like B3LYP are substantially slower and exhibit greater sensitivity to molecular structure. Notably, Molecule **2** required the least computational time across all functionals, and the ratio between the slowest and fastest functional ranged from 2.5× to 4.5× per molecule, confirming BLYP as the most computationally efficient choice overall.

## 3 Results and Discussion

### 3.1 Geometry Benchmark: GFN2-*x*TB vs B3LYP

The optimized geometries of the DS12 set obtained at the B3LYP/def2-TZVPP level are shown in Figure 1, and their corresponding molecular names (Table S1) and Cartesian coordinates are provided in the SM. Qualitatively, the semiempirical quantum mechanical GFN2-*x*TB method reproduces well the structural motifs predicted by B3LYP/def2-TZVPP for the DS12 test set. The connectivity of aromatic rings, the placement of heteroatoms, and the overall molecular shape are consistently correct. This confirms GFN2-*x*TB’s utility for rapid structure generation and conformational searching.

**Figure 1:**
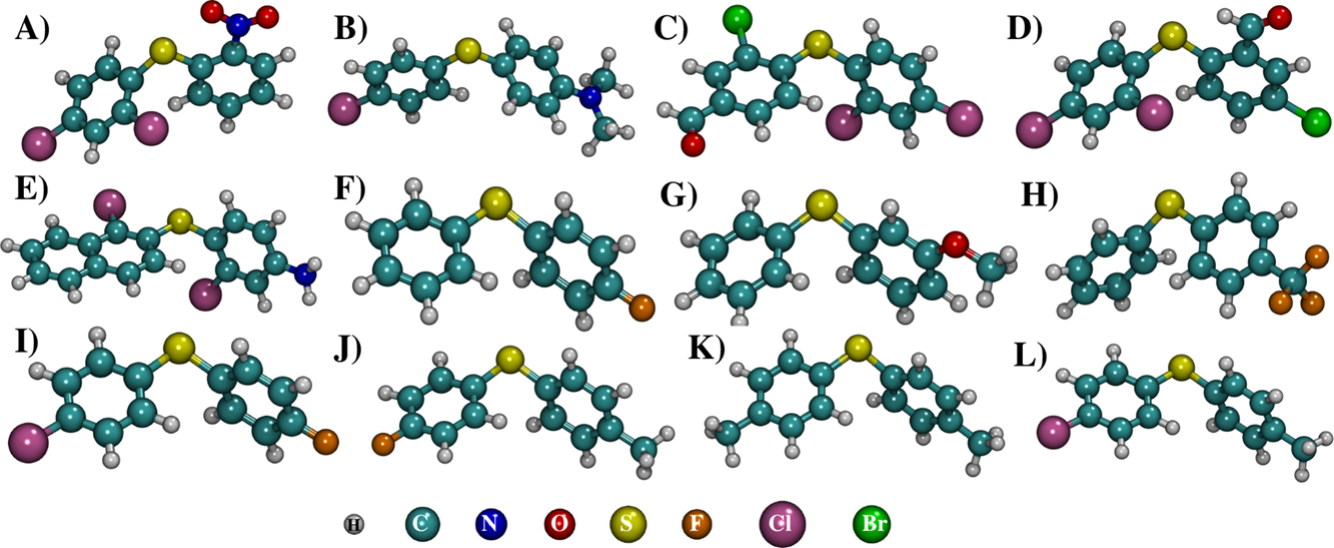
Equilibrium structures of the DS12 test set obtained at the B3LYP/def2-TZVPP level of theory. Labels (A)–(L) correspond to species **1**–**12**, respectively.

In details, deviations in bond lengths are observed across the molecular set, with the magnitude of error being highly dependent on the atom types involved. Good agreement is observed for C–C bonds within aromatic rings, with average deviations typically within 0.01–0.02 Å. For instance, in molecule **1**, aromatic C–C bonds range from 1.387 to 1.401 Å (B3LYP) versus 1.393–1.403 Å (GFN2-*x*TB). On the contrary, the most systematic differences occur in bonds involving heteroatoms, where GFN2-*x*TB consistently predicts shorter bond lengths. In molecule **1**, the C–S bond is 1.786 Å (B3LYP) vs 1.746 Å (GFN2-*x*TB), a difference of 0.04 Å and error of ∼2%. This trend is consistent across the data set (e.g., molecules **2**, **5**, and **6**). The average deviation in the C-NO_2_ bond distances is approximately 0.04–0.05 Å, representing a substantial relative error of about 3–4%. For example, in molecule **1**, the C–NO_2_ bond lengths are 1.462 Å (B3LYP) and 1.416 Å (GFN2-*x*TB), yielding a deviation of Δ*r* = −0.046 Å. For molecule **2**, the values are 1.376 Å (B3LYP) and 1.370 Å (GFN2-xTB), with a deviation of −0.006 Å, while for molecule **5** (Figure 1 E), the corresponding bond lengths are 1.383 Å (B3LYP) and 1.371 Å (GFN2-*x*TB), giving a deviation of – 0.012 Å.

Concerning the C–Cl bond lengths, a consistent shortening is observed in GFN2-*x*TB structures by 0.015–0.020 Å. For example, in molecule **1** (Figure 1 A), C–Cl bonds are 1.737–1.740 Å (B3LYP) vs 1.722–1.726 Å (GFN2-*x*TB). Similarly, C–Br bonds are shorter in GFN2-*x*TB, as seen in molecule **3** (Figure 1 C) (1.911 Å B3LYP vs 1.899 Å GFN2-*x*TB).

The most dramatic differences are seen in the torsional angles of nitro groups. In molecule **1**, the O–N–O dihedral relative to the ring plane shows differences exceeding 10–20° between methods. The B3LYP values (353.04° and 173.11°) indicate a nearly coplanar arrangement with the aromatic system, whereas the GFN2-*x*TB values (172.49° and −7.55°) suggest a significantly twisted conformation for one oxygen. One can conclude that while GFN2-*x*TB provides remarkably good qualitative structures at a fraction of the computational cost of DFT, this systematic comparison reveals non-negligible quantitative differences.

#### 3.1.1 Comparative Analysis: EDG vs EWG

Table 1 presents a comparative analysis of key geometrical parameters for molecules containing electron-donating groups (EDG) and electron-withdrawing groups (EWG). Molecules **10** and **12** provide the most direct comparison between EDG and EWG effects, featuring both types of substituents in symmetric positions. In molecule **10**, the C–F (EWG) distance measures 1.352 Å while the C–CH_3_ (EDG) distance is 1.506 Å. Similarly, molecule **12** shows C–CH_3_ and C–Cl distances of 1.506 Å and 1.748 Å, respectively. These pairs clearly illustrate the distinct bonding characteristics of electron-donating versus electron-withdrawing groups.

**Table 1:** Comparison of EDG and EWG Effects on Optimized Geometries (B3LYP/def2-TZVPP)

**molecule**	**substituent *X***	**substituent *Y***	**C–X (Å)**	**C–Y (Å)**
1	Cl (EWG)	Cl (EWG)	1.738	1.740
2	CH_3_ (EDG)	CH_3_ (EDG)	1.450	1.450
3	Br (EWG)	–CHO (EWG)	1.911	1.209^a^
4	–CHO (EWG)	Br (EWG)	1.209^a^	1.908
5	Cl (EWG)	–NH_2_ (EDG)	1.749	1.383
6	F (EWG)	–H	1.348	1.083^b^
7	–OCH_3_ (EDG)	–H	1.361	1.083^b^
8	–CF_3_ (EWG)	–H	1.498^c^	1.083^b^
9	F (EWG)	Cl (EWG)	1.348	1.747
10	F (EWG)	–CH_3_ (EDG)	1.352	1.506
11	–CH_3_ (EDG)	–CH_3_ (EDG)	1.507	1.506
12	–CH_3_ (EDG)	Cl (EWG)	1.506	1.748

a C=O bond distance.

b C–H reference.

c C–CF_3_ bond.

Molecule **5** presents an interesting case of asymmetry, combining a chlorine EWG (1.749 Å) and an amino EDG (1.383 Å). The ring bearing the amino group exhibits greater planarity, consistent with the EDG’s ability to donate electron density into the π-system, while the chlorine-substituted side maintains characteristics typical of halogenated aromatics.

Molecules **3** and **4** serve as mutual inverses, demonstrating that the position of an aldehyde relative to a bromine substituent does not significantly alter the fundamental electronic effects: both show similar C=O bond distances (1.209 Å) and comparable elongation of the adjacent C–C bonds (1.476–1.485 Å).

### 3.2 Analysis of C–S–C Bond Angle

Comprehensive visualization of C–S–C bond angles across all molecules and theoretical methods are displayed in Figure 2. As can be seen, the DFT values demonstrate remarkable consistency across different molecular systems. The relatively small standard deviation of 0.54° indicates that the C–S–C bond angle remains quite stable regardless of the molecular environment, suggesting this bond angle is relatively insensitive to substituent effects in the studied systems. The DFT averages range from 102.5° for molecule **1** to 104.5° for molecule **6**, representing a total variation span of 2° between the extreme values. This narrow range demonstrates the structural consistency of C–S–C bond angles across different molecular environments. These results align with the findings of El-Hendawy et al.,^14^ who reported a similar range of bond angles for six diaryl sulfide derivatives.

**Figure 2:**
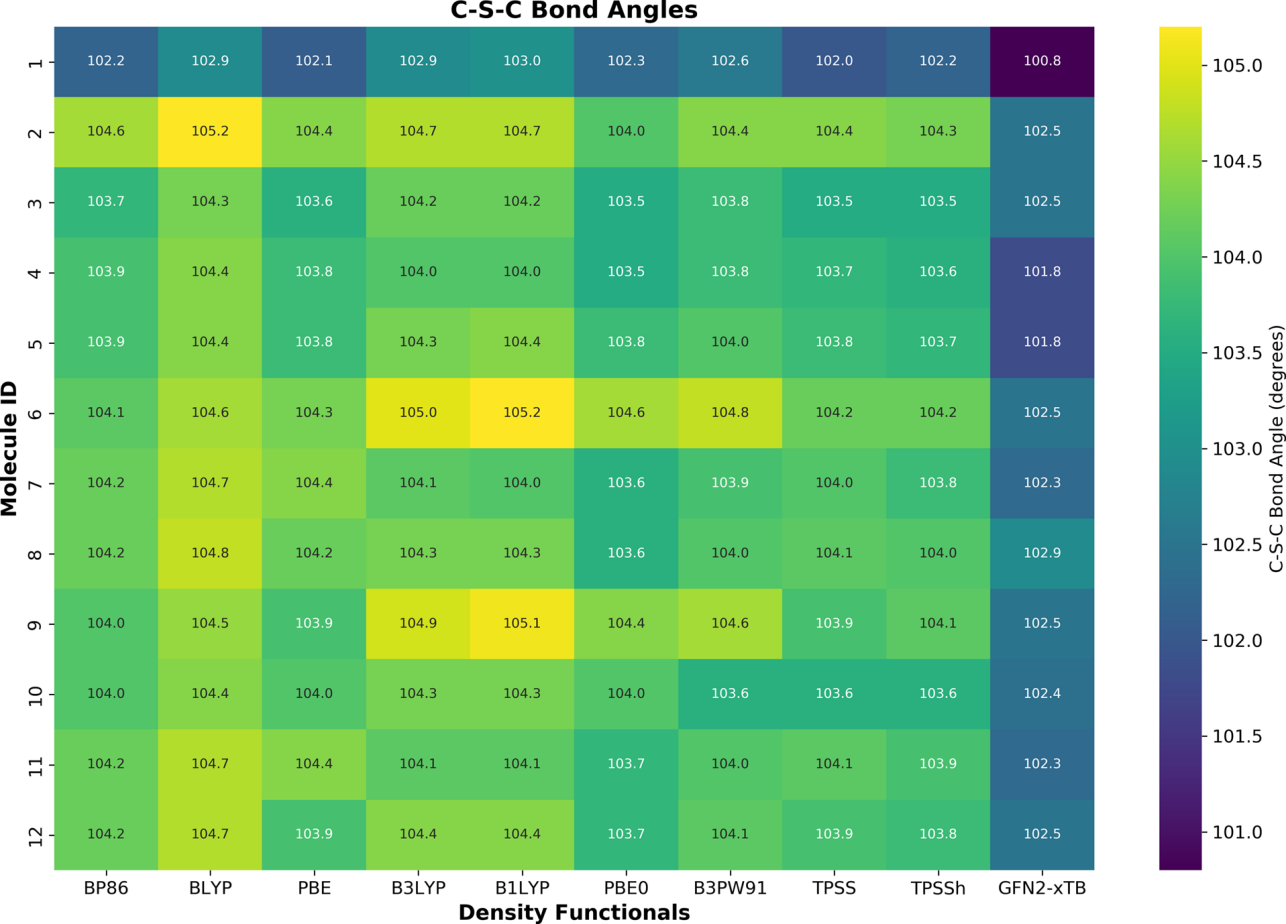
C–S–C bond angles predicted by different density functionals and GFN2-*x*TB across 12 molecular systems.

The comparative analysis between DFT functionals and the semiempirical GFN2-*x*TB approach reveals systematic trends in predicting C–S–C bond angles across 12 different molecular systems. The data demonstrate a consistent positive deviation of DFT methods relative to GFN2-*x*TB, with an average difference of ∼1.8° across all molecules. This systematic overestimation suggests that DFT functionals tend to predict larger C–S–C bond angles compared to the GFN2-*x*TB reference. For completeness, an additional plot showing this difference is presented in Figure S3 of the SM. Colored bars represent Δθ = < θ > (DFT functionals) – θ (GFN2-*x*TB) for each molecule, with color intensity proportional to deviation magnitude. The overall DFT average bond angle is 104.0 ± 0.5°, while GFN2-*x*TB yields 102.2 ± 0.5°.

### 3.3 Structural Comparison: B3LYP vs B3LYP-D3BJ

The impact of the D3BJ dispersion correction on the geometry of diaryl sulfides is not yet well established. Its inclusion leads to a systematic contraction of bond lengths, particularly for bonds involving sulfur and chlorine atoms. As an illustrative example, we consider molecule **1**. A consistent bond shortening is observed, most notably for the C–S (−0.0072 Å) and C–Cl (−0.0025 Å) bonds. Only minor variations (<1.5°) are observed for bond angles, with the largest change occurring at the C–S–C angle (−1.3°). In contrast, the most pronounced effects are found in the dihedral angles, which exhibit differences of up to approximately 8.5°. In particular, the nitro group undergoes a substantial reorientation, with dihedral angle changes of around 8°, indicating significant conformational adjustments induced by dispersion interactions.

For molecule **2**, noticeable bond contractions are observed for the S–C bond distances (from 1.7754 to 1.7709 Å, Δ = −0.0045 Å) and for the C–C bond (from 1.450 to 1.448 Å, Δ = −0.002 Å). The present findings reveal that the C–S–C bond angle is reduced by only 0.1°. For molecule **3**, the C–S bond exhibits the largest contraction, with a reduction of −0.0064 Å (from 1.7782 to 1.7718 Å). Halogen–carbon bonds also contract significantly: the C–Br bond decreases by −0.0027 Å, while the C–Cl bonds show reductions ranging from −0.0012 Å to −0.0026 Å. Additionally, the torsional angle involving the C–C–S–C moiety changes by −6.16°. In contrast, the carbonyl group (C=O) remains remarkably stable, with bond lengths and angles virtually unchanged, indicating minimal sensitivity of this functional group to dispersion effects.

For molecule **8**, the sulfur–carbon equilibrium bond distances exhibit the largest contraction, amounting to −0.004 Å, in agreement with the trends observed above. The CF_3_ group undergoes only minor angular adjustments. All three C–F bond lengths remain virtually unchanged (variations < 0.0002Å), and the F–C–F bond angles show negligible deviations (<0.07°). This pronounced structural stability contrasts with the greater flexibility of the sulfur-containing region and suggests that the highly polar, electron-withdrawing CF_3_ group is largely insensitive to dispersion-driven reorganization.

### 3.4 Mulliken Atomic Charge Analysis

Mulliken population analysis was performed with the B3LYP/D3BJ functional to investigate the charge distribution and identify potential reactive sites in the studied molecules, where significant variations in charge distribution were observed among the molecules. A detailed graphical analysis of Mulliken charges is provided in the Supporting Information (Figure S4). As can be visualized, molecule **8** exhibited the highest charge polarization, with both extremely positive (C: +0.539*e*) and negative (F atoms: ∼ −0.184*e*) sites. Molecule **1** showed typical trends for organic molecules, with oxygen atoms being strongly negative (average: −0.314*e*) and hydrogen atoms positive (average: + 0.145*e*). Molecules containing halogens (Cl, F, Br) displayed characteristic negative charges on halogen atoms, consistent with their electronegativity.

Mulliken charge analysis enabled the identification of several key reactive sites within the molecular series. Carbon centers exhibiting substantial positive charges, notably C21 in molecule **8** (+0.5395*e*) and C2 in molecule **6** (+0.2833*e*), represent potential electrophilic centers susceptible to nucleophilic attack. Conversely, oxygen atoms consistently carry significant negative charge (average: −0.290*e*), positioning them as strong hydrogen bond acceptors and potential metal coordination sites. The pronounced negative charges observed on oxygen and halogen atoms further correlate with their propensity to engage in noncovalent interactions. An exceptional case was identified in molecule **1**, where the nitrogen atom (N13) displays an unusually positive charge (+0.4349*e*), which may indicate distinctive reactivity patterns compared to conventional nitrogen centers.

As a comment, the role of the σ-hole concept in halogen bonding can be also discussed. We can define a σ-hole as a zone of positive electrostatic potential (ESP), although not always,^61^ on the bond axis, which explains the attractive interaction between electronegative halogens and electron-rich partners.^62,63^

In general, the magnitude of the σ-hole increases with the size of the halogen: *F* < Cl < Br < I. In this current work, the σ-holes in the DS12 molecules are evaluated via ESP maps calculated using the MultiWFN wave function analysis program^64^ and visualized with the VMD package.^65^ Figure 3 shown the calculated ESP maps of the species **5**, **8**, **9**, and **10** at the B3LYP-D3BJ/def2-TZVPP level of theory. The isosurfaces (0.001 au) illustrate that regions of negative electrostatic potential (shown in blue) are localized around the halogen atoms (X = F, Cl) involved in the X–C bonds. The maps suggest the presence of σ-holes on the X–C bonds, in agreement with established findings.^61^ However, its direct manifestation in a single, noninteracting (free) molecule is not as pronounced, which is the context of our primary analysis.

**Figure 3:**
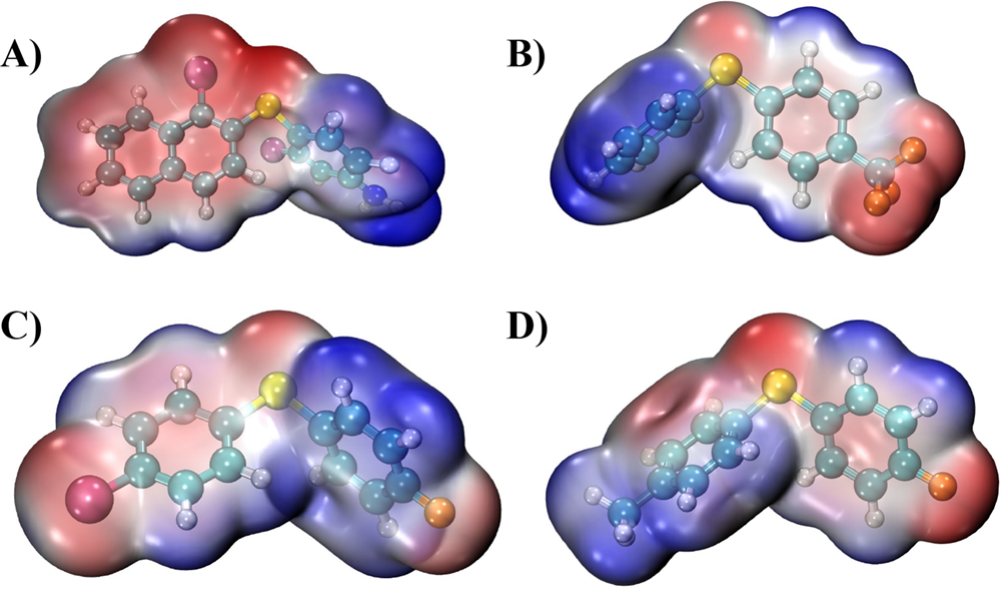
3D plots of electrostatic potential (ESP) map calculated at B3LYP/D3BJ/def2-TZVPP level of theory for compounds (A) **5**, (B) **8**, (C) **9**, and (D) **10**. The ESP is projected in atomic units onto the 0.001 au electron density isosurface. Regions of positive ESP (electrophilic) are shown in blue, while regions of negative ESP (nucleophilic) are shown in red. Color coding is consistent with Figure 1.

### 3.5 Analysis of Dipole Moment Accuracy across Computational Methods

The numerical values of dipole moment, μ, calculated for the DS12 test set using different DFT functionals and the high-level DLPNO–CCSD­(T) method are presented in Table S2 in the SM. The values span a considerable range, from a minimum of approximately 0.78–1.16 D for molecule **6** to a maximum of approximately 5.82–5.89 D for molecules **2** and **5**. We can attribute these exceptionally high values signal a pronounced charge separation across the molecule. This is typically achieved by a “push–pull” architecture: one aryl ring is substituted with a strong electron-donating group (EDG), which raises electron density on that ring (partial negative charge, δ^–^), while the other ring bears a strong electron-withdrawing group (EWG), which depletes electron density (partial positive charge, δ^+^). The sulfur bridge then connects these two oppositely charged moieties, creating a large dipole moment along the long molecular axis (see Figures 1 and 3).

This observed range, within the interval μ ∈ [1.0,6.0] D, is consistent with the typical magnitudes suggested for diaryl sulfide compounds, as previously discussed.^66,67^ For instance, the calculated dipole moment of 2-isopropylphenyl 2-nitro-4-((E)-((4-acetylpiperazin- 1-yl)­carbonyl)­ethenyl)­phenyl sulfide^66^ (6.09 D using B3LYP) falls near the upper end of this distribution, closely aligning with the values computed for the most polar derivatives in the present series, such as **1** (5.03 D with B3LYP) and **2** (5.60 D with B3LYP). Moreover, the dipole moments calculated for diphenyl sulfone and diphenyl sulfoxide, which possess analogous geometrical structures, have been reported to be larger, in the range of 6.4–14.4 D.^14^

Table 2 shows the average dipole moments across DS12 set. The consistency of computational predictions varies substantially across the molecular set, with molecule **12** demonstrating the most reliable predictions across methods (Std. Dev. = 0.14 D), whereas molecule 6 shows the poorest agreement among methods (Std. Dev. = 0.40 D). Molecule **6** exhibits remarkably low polarity (0.98 D), which can be attributed to its specific molecular geometry. The opposing positions of the strongly electron-withdrawing fluorine and sulfur atoms create a scenario where the two strong electron-withdrawing groups largely cancel each other’s dipole contributions, resulting in the observed low net polarity.

**Table 2:** Average Dipole Moments for All Molecules Calculated across Different Computational Methods

molecule	average dipole (D)	std. dev. (D)
1	5.07	0.30
2	5.69	0.19
3	2.98	0.22
4	4.71	0.32
5	5.69	0.16
6	0.98	0.40
7	2.60	0.20
8	3.91	0.18
9	1.19	0.21
10	2.29	0.35
11	2.00	0.21
12	3.26	0.14

The generally good agreement between different DFT functionals for most molecules, and their reasonable proximity to the reference DLPNO–CCSD­(T) values where available, reinforces the reliability of these computational approaches for property prediction in diaryl sulfides. These results are presented in Figure 4. The color intensity from yellow to red indicates increasing magnitude of deviation, highlighting method-molecule combinations with largest discrepancies. The statistical analysis reveals a clear hierarchy in the accuracy of dipole moment predictions relative to the DLPNO–CCSD­(T)/cc-pVTZ reference method. For the whole set, B1LYP emerges as the most accurate functional with the lowest Mean Absolute Error (MAE = 0.246 D) and Root Mean Square Error (RMSE = 0.283 D), closely followed by B3LYP (MAE = 0.262 D, RMSE = 0.302 D), see also Figure S5 in the SM. This superior performance of hybrid functionals containing exact Hartree–Fock exchange suggests that the inclusion of nonlocal exchange correlation is crucial for accurate dipole moment predictions.

**Figure 4:**
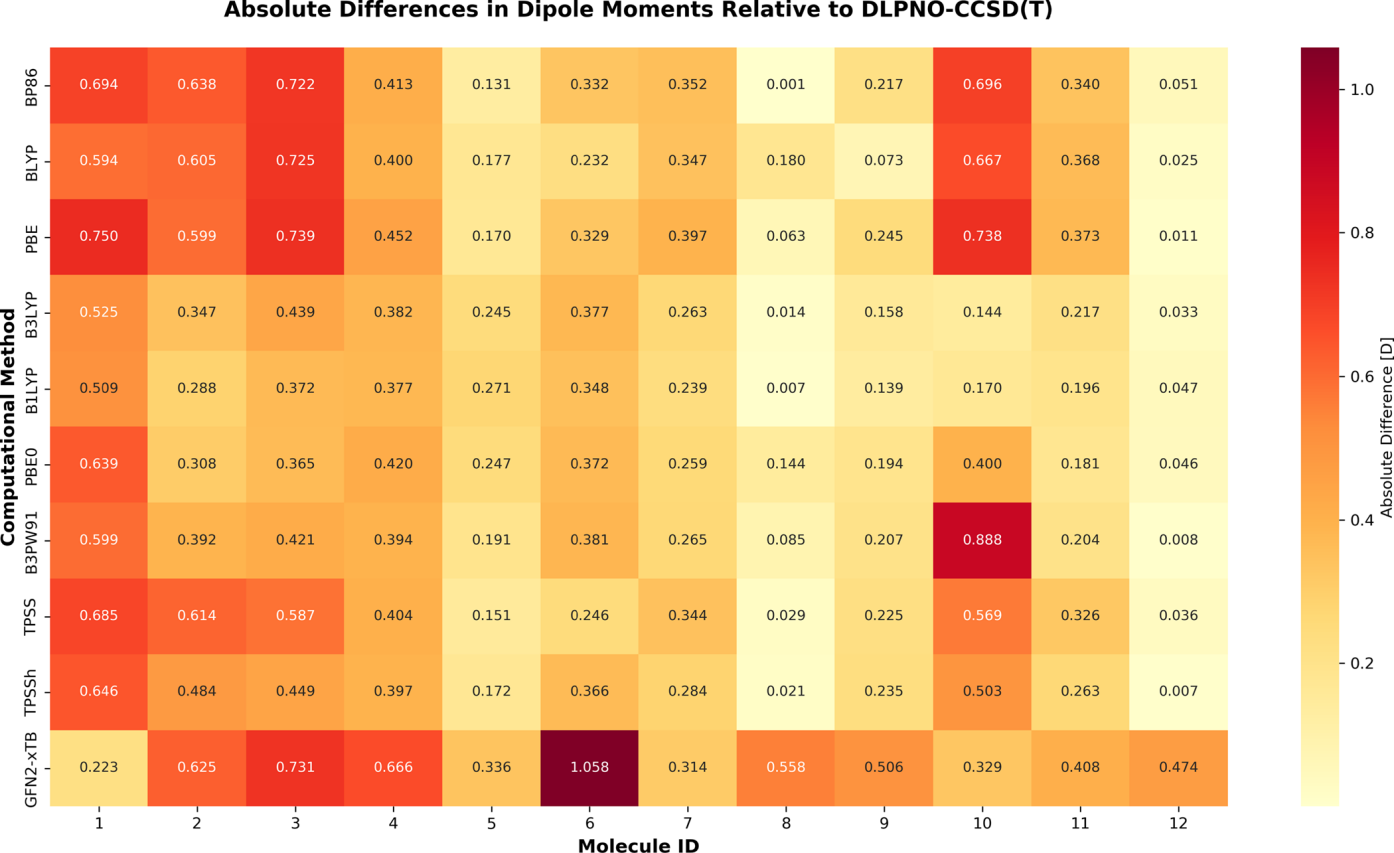
Absolute deviations in dipole moment calculations relative to DLPNO–CCSD­(T) reference values. The color gradient from yellow to red represents increasing absolute deviation in Debye (D), with GFN2-*x*TB showing the most significant errors (particularly for molecule 6:1.058 D) while hybrid functionals like B3LYP and PBE0 demonstrate smaller deviations for most molecules.

The middle-performance group consists of PBE0 (MAE = 0.297 D), TPSSh (MAE = 0.318 D), and B3PW91 (MAE = 0.336 D), all hybrid or meta-hybrid functionals. The generalized gradient approximation (GGA) functionals BLYP, BP86, and PBE show moderate performance with MAE values of 0.366, 0.382, and 0.405 D, respectively. Notably, GFN2-*x*TB, a semiempirical method, demonstrates the poorest performance (MAE = 0.519 D), indicating limitations in describing electrostatic properties despite its computational efficiency.

All DFT functionals exhibit a consistent negative mean error, ranging from −0.098 D (BLYP) to −0.166 D (B3PW91), indicating a systematic underestimation of dipole moments compared to the high-level reference. This systematic bias suggests that current density functionals tend to delocalize electron density excessively, reducing the charge separation and consequently the dipole moments.

For researchers requiring accurate dipole moment predictions, B1LYP and B3LYP offer the best compromise between accuracy and computational cost. In contrast, the poor performance of GFN2-*x*TB for dipole moments cautions against its use for properties highly sensitive to electron distribution, despite its advantages in geometry optimization and energetic calculations. The molecular dependence of the errors further highlights the importance of method validation for specific chemical systems. Interestingly, a more detailed analysis reveals that, although the GFN2-*x*TB method presents such issues, it can sometimes provide dipole moment values that are closer to the coupled-cluster results than those obtained with other DFT functionals.

The correlation between DFT ensemble averages taken from Table 2 and DLPNO–CCSD­(T) reference values shows excellent agreement across all 12 molecular systems studied (Figure 5). The linear regression analysis yields the relationship:

μ(x)=0.986x+0.115 6

with a Pearson correlation coefficient of *R* = 0.983 (*R* ^2^ = 0.966). The MAE of 0.251 D and RMSE of 0.299 D demonstrate good quantitative agreement. The maximum absolute deviation is observed for molecule **1** (Δ = −0.488 D), while the minimum deviation occurs for molecule **12** (Δ = −0.047 D). The mean absolute percentage error across all molecules is 4.73%, with individual errors ranging from 0.36% to 13.26%.

**Figure 5:**
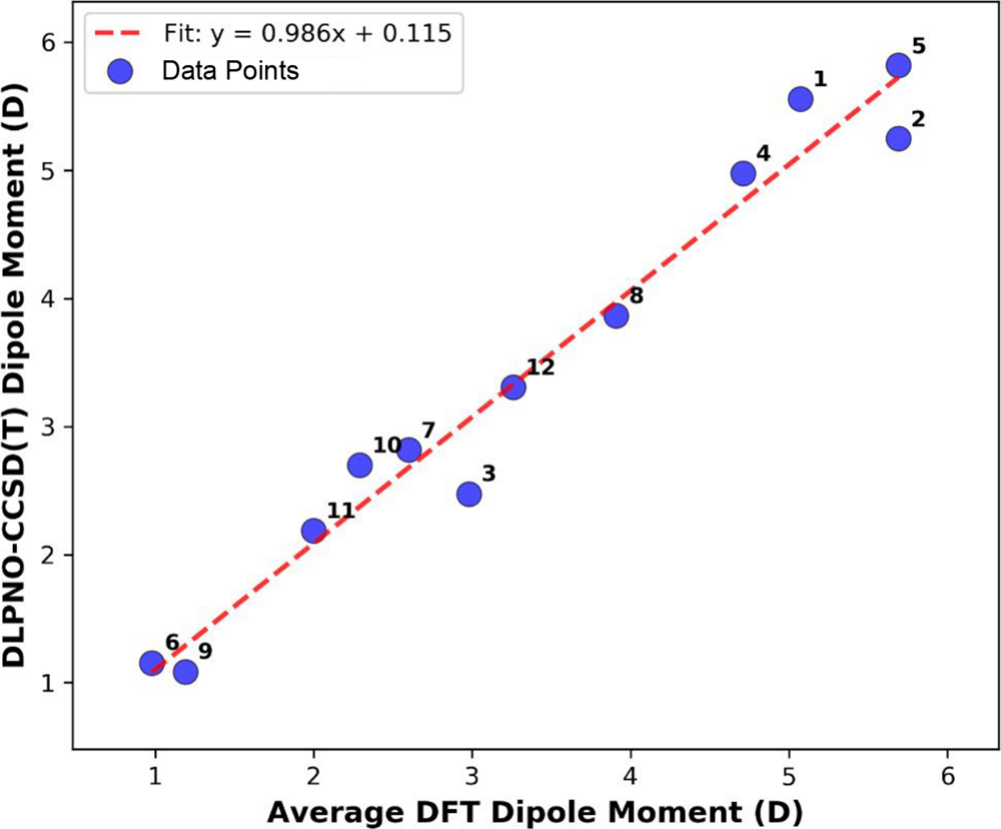
Correlation analysis between DFT ensemble averages and DLPNO–CCSD­(T) reference dipole moments. Linear correlation plot showing the strong relationship between methods (*R* = 0.983). The red dashed line represents the linear regression fit, with the corresponding equation indicated in the legend. Each data point is labeled with the corresponding molecule ID.

In addition, the comparative analysis of dipole moments calculated at the DLPNO–CCSD­(T) and GFN2-*x*TB levels for the set of 12 molecules displayed in Figure S6 of Supporting Information reveals a complex picture of performance, characterized by a strong overall correlation but significant specific discrepancies. The most striking initial observation is the strong positive correlation between the two methods, evidenced by a correlation coefficient (*R*) of 0.97 and a coefficient of determination (*R* ^2^) of 0.93. This indicates that GFN2-*x*TB can eventually captures the general trends in dipole moment variation across the molecular series.

### 3.6 Evaluation of Ionization Potentials

This study presents a comprehensive analysis of ionization potentials, IPs, obtained from DFT calculations. The calculated IPs for all 12 molecules, evaluated different DFT functionals and coupled-cluster method, are presented in Table S3 of the SM. Average values are summarized in Table 3. As results, hybrid functionals tend to predict higher ionization potentials compared to pure GGA functionals. This systematic difference is primarily attributed to the self-interaction error (SIE) inherent to local and semilocal functionals.^68,69^ GGA functionals lack exact exchange, which leads to an incomplete cancellation of the electron’s self-Coulomb interaction. Consequently, the electron density becomes excessively delocalized, resulting in an overestimation of the highest occupied molecular orbital energy and, hence, an underestimation of the ionization potential. The incorporation of exact exchange in hybrid functionals mitigates this error, yielding more accurate IPs.

**Table 3:** Average Ionization Potentials

molecule	average IP (eV)	std. dev. (eV)
1	7.771	0.137
2	6.417	0.112
3	7.789	0.138
4	7.711	0.140
5	6.754	0.129
6	7.326	0.125
7	7.133	0.130
8	7.608	0.123
9	7.306	0.119
10	7.153	0.113
11	6.957	0.109
12	7.156	0.118

Based on the average ionization potentials, molecules **1**, **3**, and **4** exhibit high IP values (>7.6 eV), molecules **6**, **8**, **9**, **10**, and **12** fall into a medium range (7.0–7.6 eV), while molecules **2**, **5**, **7**, and **11** are characterized by low IPs (<7.0 eV). Among then, molecule **3** has the highest IP numbers, ranging from 7.5 to 8.0 eV, while molecule **2** has the lowest IP (6.2–6.6 eV).

Figure 6A presents a plot of the signed errors between the DFT-calculated and coupled-cluster reference IP values. From a statistical perspective, the PBE0 and B3PW91 functionals demonstrate exceptional accuracy, with MAE below 0.060 eV (see Figure S7). Both functionals incorporate approximately 25% exact exchange and exhibit minimal systematic bias, with PBE0 showing a slight tendency toward overestimation and B3PW91 displaying comparable behavior. Their robustness across diverse molecular systems is further supported by low standard deviations of 0.078 and 0.080 eV, respectively.

**Figure 6:**
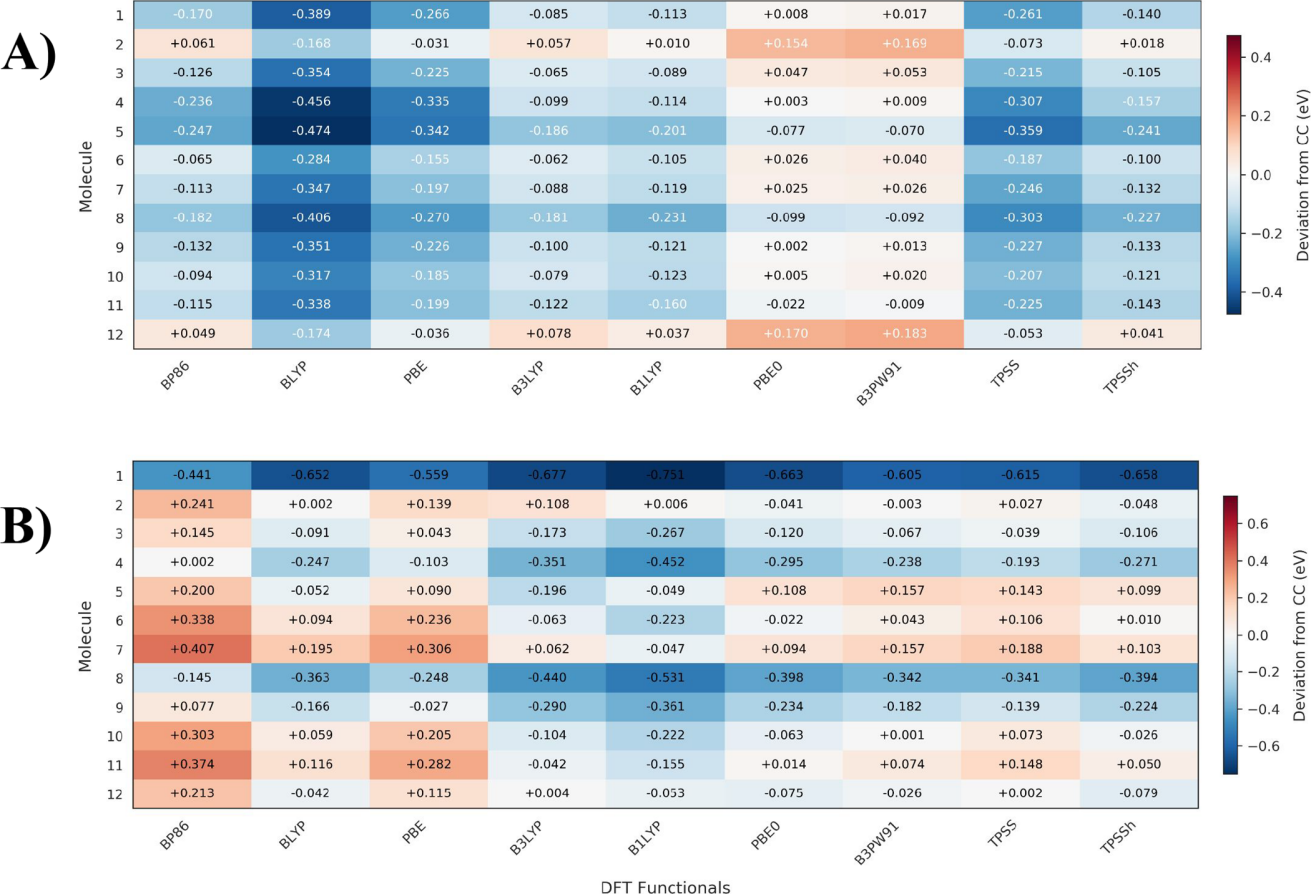
Signed errors (DFT - CC) for (A) Ionization Potential and (B) Electron Affinity, showing systematic underestimation (blue) or overestimation (red) relative to the coupled-cluster (CC) reference.

In contrast, B3LYP (MAE = 0.100 eV) and B1LYP (MAE = 0.118 eV) show moderate systematic underestimation of IP values. BLYP performs particularly poorly, exhibiting a pronounced systematic underestimation of −0.338 eV, which renders it unsuitable for applications requiring high quantitative accuracy.

Additionally, the DFT-computed dipole moments for cationic species (Table S4, SM) reveal that molecule **2** has the highest values and molecule **11** the lowest across all functionals. Significant functional dependence is evident, with maximum percentage deviations of 21.7% (molecule 2, B1LYP vs PBE), 28.1% (molecule 9, B1LYP vs PBE), and 18.3% (molecule 11, BLYP vs B1LYP).

### 3.7 Evaluation of Electron Affinities

The present analysis of the thermochemical properties of the DS12 set is further extended to electron affinities, EAs, which were calculated using additional diffuse functions within the def2-TZVPP basis set. Accurate prediction of electron affinities remains a fundamental challenge in computational quantum chemistry, with important implications for a wide range of applications, including charge-transfer processes.^70^ The computed numbers are listed in Table S5 of the SM.

Figure 6B presents the signed errors between the DFT-calculated and coupled-cluster reference EA values. Statistically, the B3PW91 functional exhibits the best overall performance, yielding a mean absolute error (MAE) of 0.158 eV, as shown in Figure S7, and minimal systematic bias, as indicated by a mean deviation of −0.086 eV. Notably, the meta-GGA functional TPSS also demonstrates strong predictive capability, with an MAE of 0.168 eV.

In contrast, B3LYP shows only moderate accuracy, with an MAE of 0.209 eV and pronounced systematic underestimation (−0.180 eV). B1LYP performs the worst among the tested functionals, exhibiting the largest overall errors (MAE = 0.259 eV) and a significant negative bias (−0.259 eV).

### 3.8 DFT Functional Performance for Vertical Transition Energies

We performed a comprehensive statistical analysis to assess the performance of nine density functional theory functionals in calculating vertical transition energies across DS12 test set computed as

$$E^{\mathrm{vert}}=E^{\mathrm{ES}}(R^{\mathrm{GS}})-E^{\mathrm{GS}}(R^{\mathrm{GS}})$$ 7

where *R* ^GS^ represents the ground state geometry, and *E* ^ES^ and *E* ^GS^ correspond to the excited and ground state energies, respectively. In the absence of experimental measurements for direct benchmarking, we adopted the highly correlated STEOM-DLPNO–CCSD/cc-pVTZ level of theory for additional validation. This analysis provides important insights into the behavior of different functionals and offers quantitative guidance for the selection of reliable methodologies in TDDFT studies. The TDDFT/TDA data obtained are provided in Table S6 of the SM. Additional results obtained using the D3BJ dispersion correction were also included. Additionally, the primary orbital configurations for the lowest singlet (S_1_) and triplet (T_1_) excited states are tabulated in Table S7 of the SM.

The present findings reveal that molecule **1** exhibits the lowest S_0_–S_1_ transition energies (2.837 to 3.631 eV) among the series, characterized as a π → π∗ transition, with moderate functional sensitivity (0.794 eV range) and modest oscillator strengths (*f* ≈ 0.1–0.3). Its relatively small S_1_–T_1_ energy gap (0.536–0.718 eV) suggests significant singlet–triplet mixing potential. In contrast, molecule **2** represents a high-sensitivity case, with S_0_–S_1_ energies spanning 0.887 eV (3.560–4.447 eV); this substantial functional dependence reflects pronounced differences in how various functionals describe its excited-state electronic structure.

In general, molecules **4** and **12** without strong electron-withdrawing groups also present π → π∗ transitions. Analysis of Table S5 reveals a clear trend: molecules **2**, **7**, and **11** demonstrate the highest sensitivity, with S_1_ energy variations exceeding 0.85 eV, whereas molecules **1**, **4**, and **12** yield more consistent results across functionals, with variations below 0.75 eV. The vertical S_1_–T_1_ energy gap plays a crucial role in studying the intersystem crossing process within a Jablonski diagram. Therefore, the corresponding values for the DS12 set are provided in Figure S8 of the SM.

Table S7 in the Supporting Information reveals that, for the majority of compounds, both the S_1_ and T_1_ excited states are predominantly described by a single dominant transition. In particular, the HOMO → LUMO (H→L) transition constitutes the major contribution for most S_1_ states (compounds **1, 5, 7, 10, 11, 12** with >90%) and many T_1_ states (compounds **1, 2, 3, 5, 6, 7, 10, 11**). This pattern is characteristic of locally excited (LE) states in conjugated organic systems and suggests that the frontier molecular orbitals are appropriately positioned to describe the lowest-energy excitations.

However, notable exceptions and variations provide insight into subtler electronic effects. In several instances (e.g., compounds **4, 5, 7**), the T_1_ state exhibits significantly greater multiconfigurational character than its corresponding S_1_ state. For example, in compound **5**, the S_1_ state is almost purely H→L (98%), while the T_1_ state includes substantial contributions from H–1 → L (10%) and H–2 → L (5%). This increased configuration mixing in the triplet manifold may influence intersystem crossing rates and triplet-state relaxation pathways, as the involvement of multiple orbital transitions can affect spin–orbit coupling matrix elements and vibronic coupling.

The performance of the DFT methods is quantitatively assessed in Figure 7, which plots the signed errors for singlet–singlet (Panel A) and singlet–triplet (Panel B) vertical excitations against the STEOM-DLPNO–CCSD/VTZ reference. As shown, a predominant blue coloration indicates a systematic underestimation of excitation energies, whereas red bars denote overestimation.

**Figure 7:**
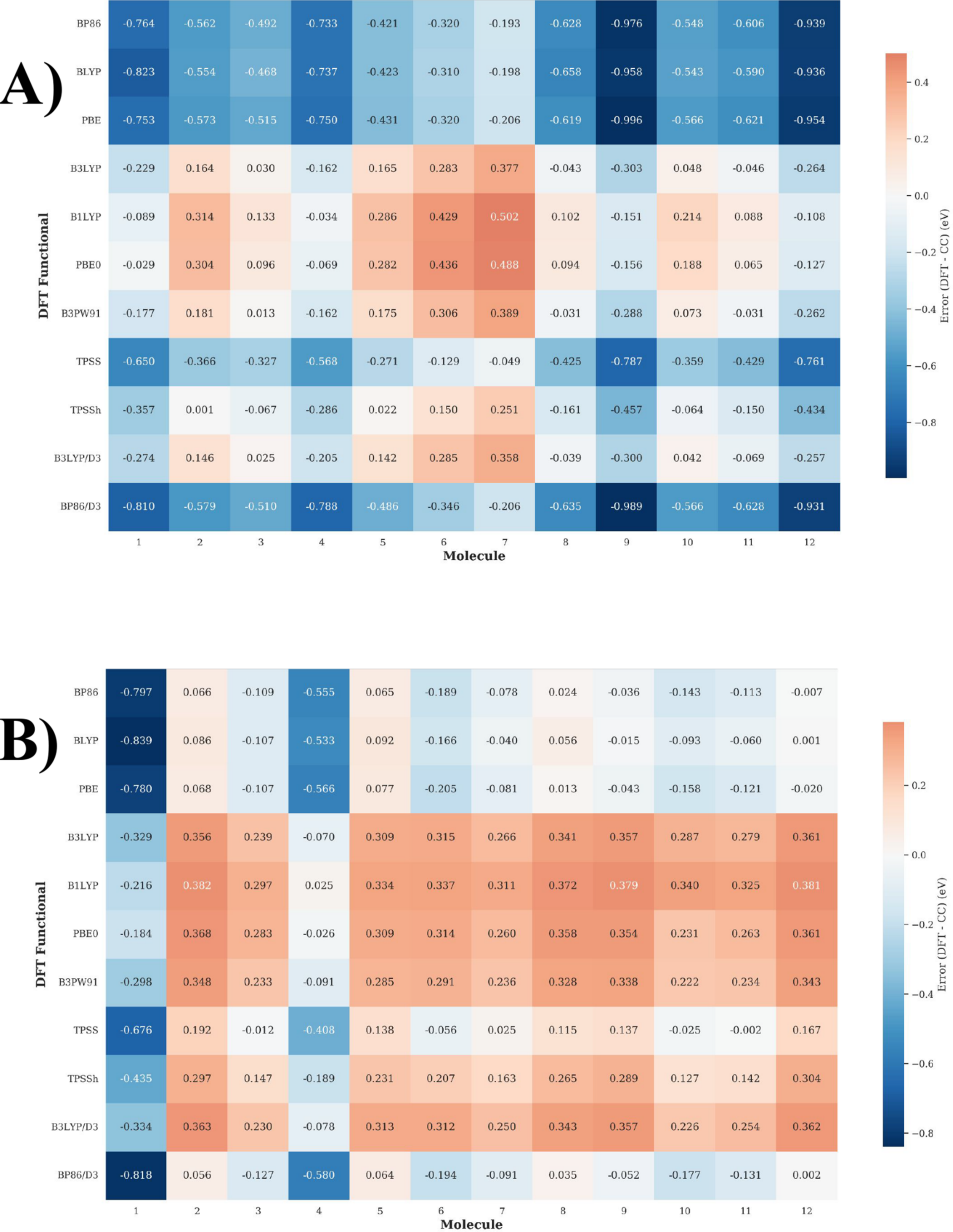
Signed errors (TDDFT/TDA - CC) for A) S_0_ → S_1_ and B) S_0_ → T_1_ vertical energies, showing systematic underestimation (blue) or overestimation (red) relative to the STEOM-DLPNO–CCSD reference. Data plotted from Table S3 in the SM. For S_0_ → S_1_ transitions, pure GGA functionals (BP86, BLYP, PBE) consistently underestimate excitation energies by 0.2–1.0 eV, while hybrid functionals (B3LYP, PBE0, B1LYP) show mixed behavior with both overestimations and underestimations. Dispersion corrections have minimal impact on signed errors compared to their uncorrected counterparts.

For singlet–singlet vertical energy transitions, GGA functionals exhibit systematic underestimation of excitation energies when compared to coupled-cluster results, with errors ranging from −0.193 eV to −0.996 eV. BP86, BLYP, and PBE show average errors of −0.620 eV, −0.619 eV, and −0.625 eV, respectively. The addition of D3 dispersion correction to BP86 results in nearly identical errors (−0.635 eV), indicating negligible improvement for singlet excitations.

Hybrid functionals show mixed error patterns with both positive and negative deviations, ranging from −0.303 eV to +0.502 eV. B3PW91 exhibits the most balanced performance with maximum errors of ±0.389 eV. The similarity between B3LYP and B3LYP/D3 error patterns confirms that dispersion corrections provide minimal benefit for singlet excitation energies.

Significant molecular dependence is observed in DFT performance. Molecules **9**, **12**, and **1** show the largest errors compared to singlet–singlet transition energies calculated employing the STEOM-DLPNO–CCSD method, with GGA functionals underestimating by up to −0.996 eV. Conversely, molecules **7**, **6**, and **3** demonstrate much better agreement, with errors as low as −0.193 eV for BP86 on molecule **7** and nearly perfect predictions from B3PW91 on molecule 3 (+0.013 eV).

For S_0_ → T_1_ transition energies, the present analysis reveals that GGA functionals show mixed errors (−0.839 eV to +0.092 eV) with predominant underestimation, particularly for molecule **1**. Hybrid functionals systematically overestimate triplet energies (−0.329 eV to +0.382 eV), with B1LYP showing the largest overestimations and B3PW91 providing the most balanced performance. Meta-GGA functionals exhibit intermediate behavior, with TPSS showing mixed errors and TPSSh demonstrating generally positive errors and improved accuracy over TPSS. In addition, TPSSh outperforms other functionals, unlike for singlets where hybrids dominate. As can be observed, D3 corrections provide slight improvement for both transition types.

The performance metrics of the nine DFT functionals for calculating vertical transition energies to the first singlet (S_1_) and triplet (T_1_) excited states show that the hybrid-meta GGA TPSSh demonstrated superior performance with the lowest mean absolute errors (MAE = 0.101 eV for singlet–singlet, 0.063 eV for singlet–triplet), establishing it as the most accurate method among those tested. The meta-GGA TPSS and hybrid GGA B3LYP and B3PW91 showed competitive accuracy among conventional functionals. The improvement offered by TPSSh compared to TPSS can be attributed to the inclusion of ∼10% exact exchange in its formulation.^43^

Based on these statistical findings, the TPSSh or B3PW91 functionals are recommended for exploratory calculations requiring reliable vertical transition energies in diaryl sulfides. This conclusion is consistent with the comprehensive review by Suellen et al.,^71^ who reported an RMSE of 0.29 eV for TPSSh when investigating a set of 41 electronic transitions in small- and medium-sized organic molecules. However, it is worth noting that TPSSh has been reported to yield some of the poorest singlet–triplet gap predictions for the AC-18 data set previously analyzed by Shirazi et al.^51^ For the present systems, hybrid functionals generally outperform other functionals for vertical transition energy calculations, with the inclusion of exact exchange.

#### 3.8.1 TDA vs Full TDDFT: A Comparative Assessment

To evaluate the impact of the Tamm-Dancoff approximation on our calculated vertical excitation energies, we performed selected calculations using the full TDDFT formalism (i.e., without TDA) for all 12 molecules in the DS12 test set. These calculations were carried out using four representative functionals: B3LYP, B3LYP/D3BJ, B3PW91, and TPSSh. The results are presented in Table S8, alongside the corresponding TDA values from our main benchmark study.

The comparison reveals excellent agreement between TDA and full TDDFT for singlet–singlet transitions (ΔE^*S*_0_→*S*_1_^), with MAE of only 0.035, 0.032, 0.036, and 0.034 eV for B3LYP, B3LYP/D3BJ, B3PW91, and TPSSh, respectively. For triplet excitations (ΔE^*S*_0_→*T*_1_^), the deviations are slightly larger but still modest, with MAE of 0.112, 0.109, 0.124, and 0.104 eV for the same functional order.

Molecules with strong electron-withdrawing groups (e.g., **1**, **8**, **9**) show excellent agreement for both singlet and triplet transitions, with deviations consistently below 0.1 eV. In contrast, molecules **2**, **5**, and **7** exhibit slightly larger deviations, particularly for triplet states. Notably, molecule **5** shows the largest TDA overestimation for triplet excitations across all functionals (0.23–0.25 eV), suggesting that the electronic structure of this specific derivative may be more sensitive to the TDA approximation.

### 3.9 Comparative Analysis of Frontier Molecular Orbital Properties

The calculated HOMO and LUMO orbitals of the diaryl sulfide derivatives are shown in Figure 8, and their corresponding numerical values are listed in Table S9 of the Supporting Information. The HOMO–LUMO energy gap exhibits substantial variation across the molecular series. Accordingly, the HOMO–LUMO energy gaps of the DS12 molecules, calculated using different DFT functionals, are depicted in Figure 9 and Table S10. In addition, all global reactivity descriptor values are provided in the Supporting Information files. A summary of the main results is presented in Figure 10.

**Figure 8:**
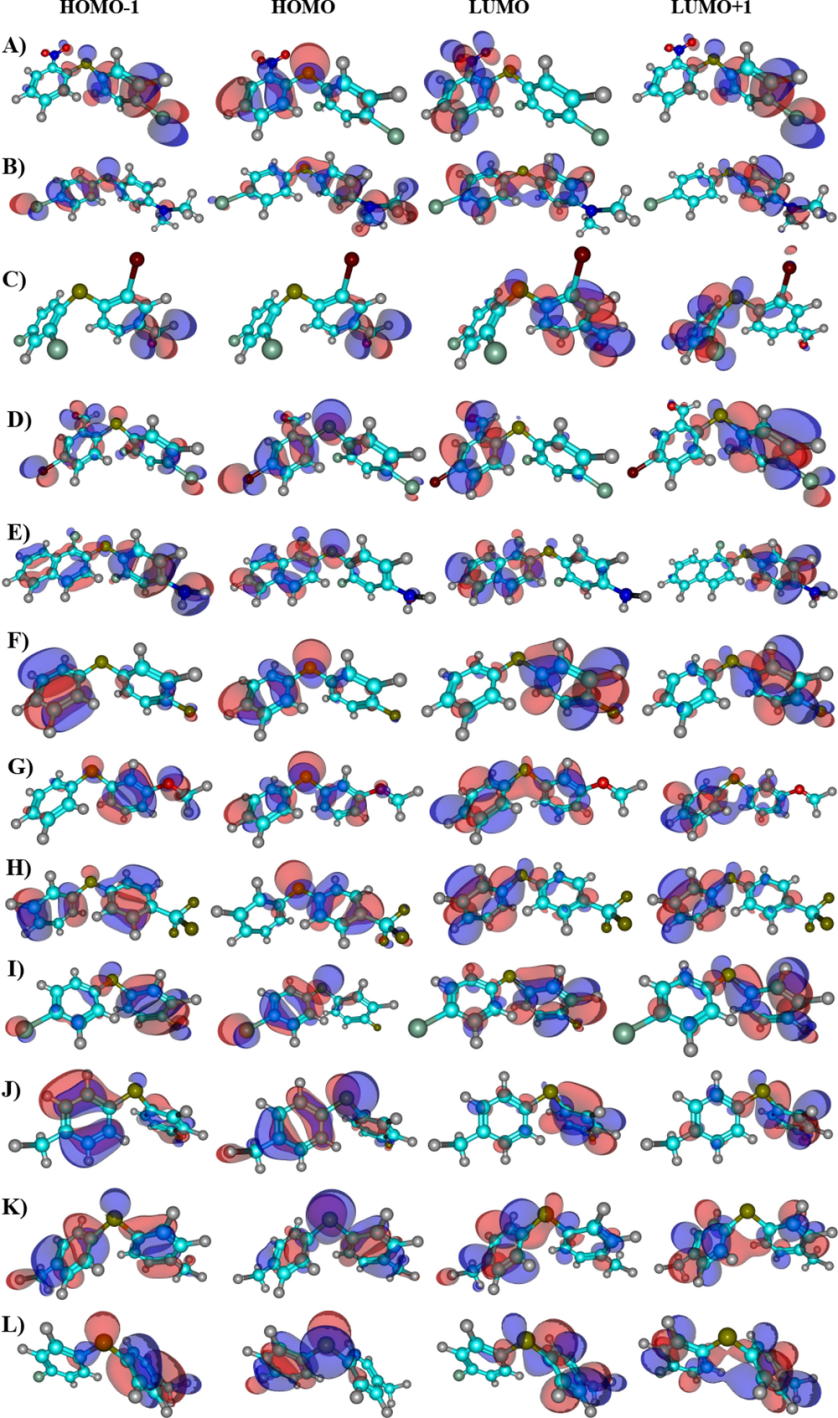
Representation of the frontier molecular orbitals (HOMO–1, HOMO, LUMO, and LUMO+1.) obtained from B3LYP/def2-TZVPP calculations:: (A) **1**, (B) **2**, (C) **3**, (D) **4**, (E) **5**, (F) **6**, (G) **7**, (H) **8**, (I) **9**, (J) **10**, (K) **11**, (L) **12**. (Atom labels: olive = sulfur, light blue = carbon, white = hydrogen, red = oxygen, blue = nitrogen, green = chlorine). The isosurface value was set to 0.02 atomic units for optimal visualization of nodal patterns.

**Figure 9:**
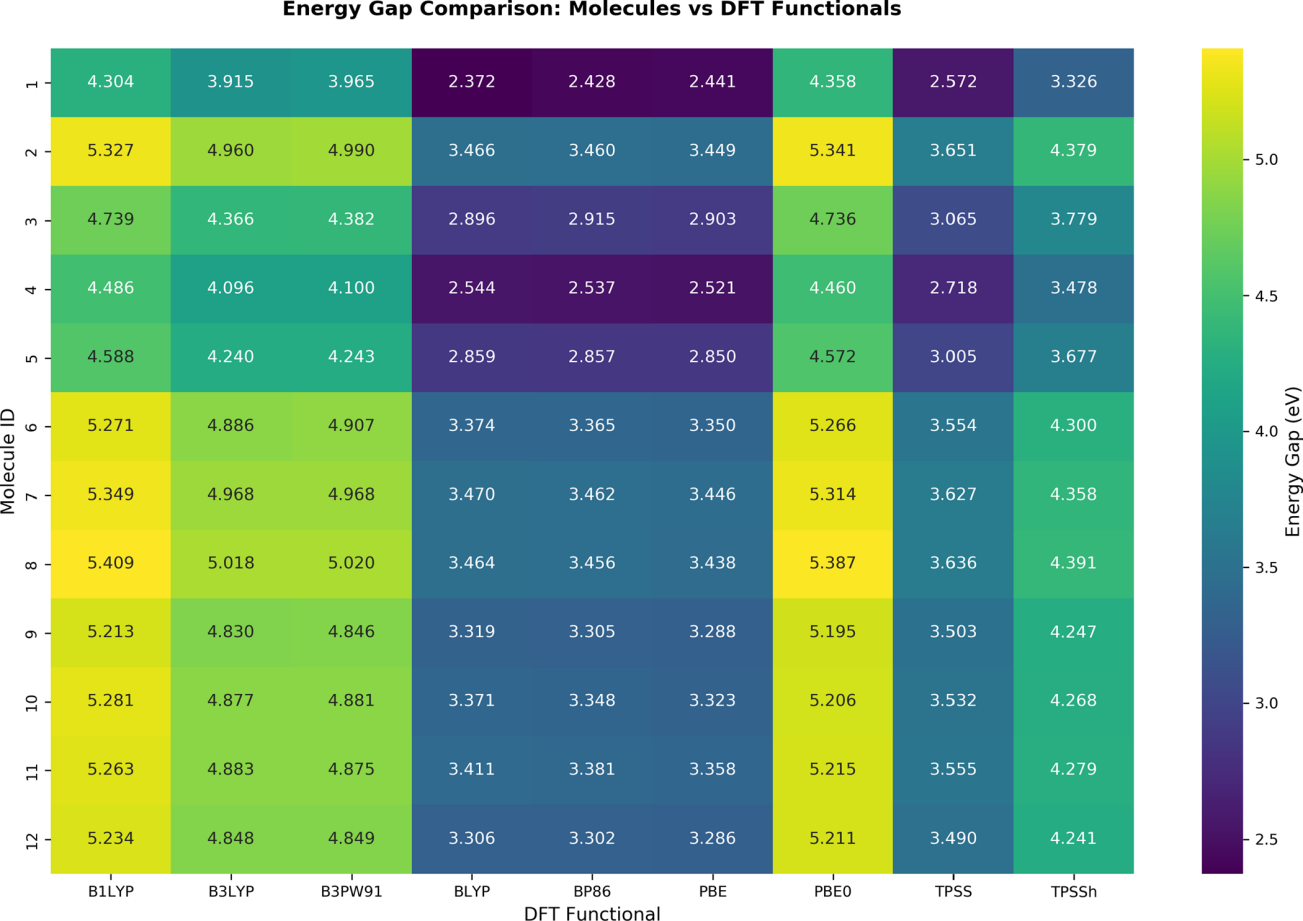
HOMO–LUMO energy gaps (in eV) for 12 molecules calculated with 9 different DFT functionals. The color intensity represents the magnitude of the energy gap, with lighter colors indicating larger gaps and darker colors indicating smaller gaps.

**Figure 10:**
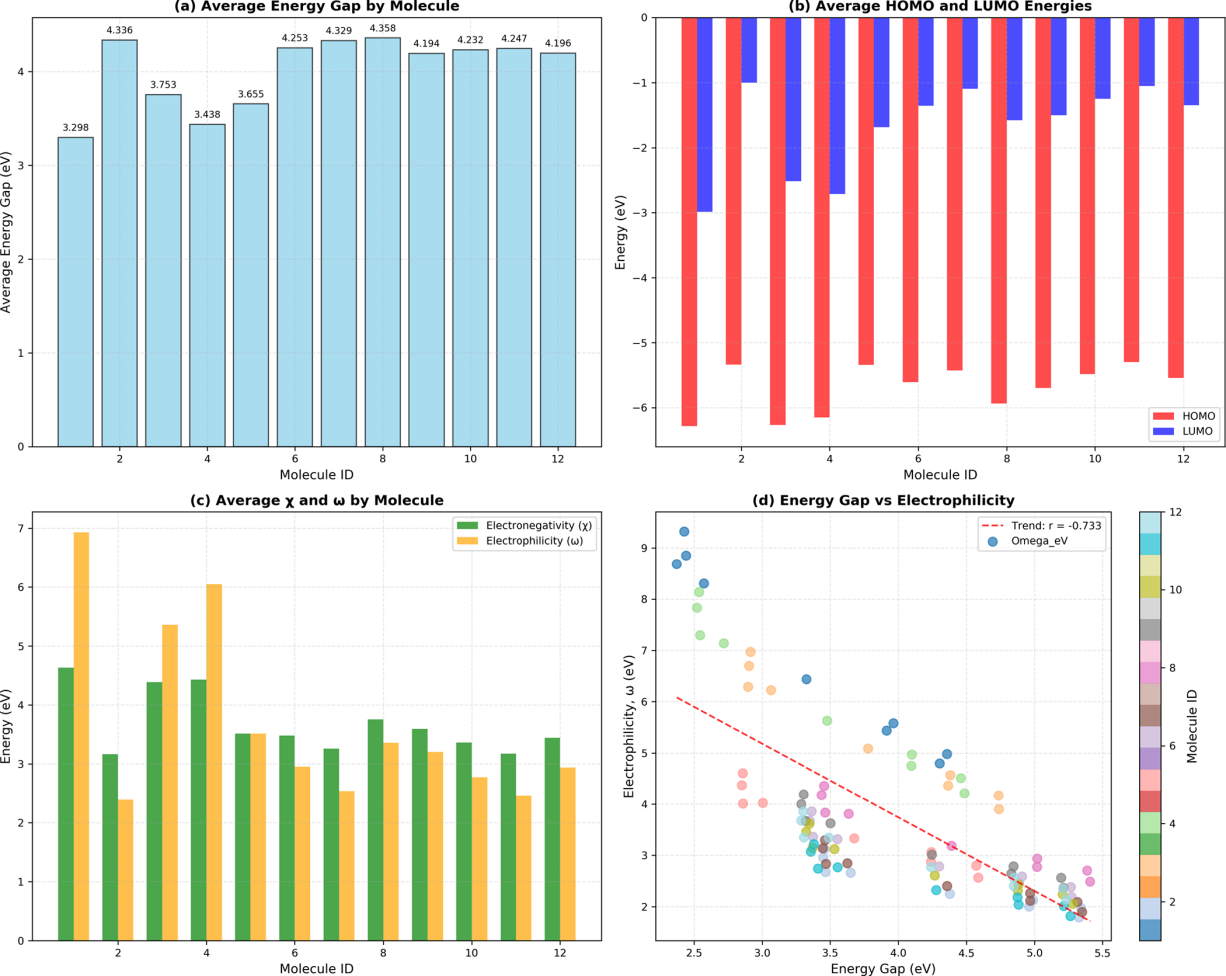
Comparative analysis of electronic properties across 12 molecules: (a) average energy gap by molecule, (b) Comparison of average HOMO and LUMO energy levels, (c) Electronegativity (χ) and electrophilicity (ω) values, and (d) Correlation between energy gap and electrophilicity, colored by molecule ID.

The distinctive electronic behavior of diaryl sulfides is fundamentally rooted in the interaction between the sulfur atom’s lone pairs and the aromatic π-systems. This interaction enables the sulfur to act as an electronic bridge, facilitating delocalization across the entire molecular framework. The composition of the frontier molecular orbitals, particularly the HOMO, reflects this. As visualized in Figure 8, the HOMO is not localized on a single ring but is distributed across both aryl moieties with a significant contribution from the sulfur atom. This extended conjugation renders the electronic properties, such as the HOMO–LUMO gap, dipole moment, and excitation energies, highly sensitive to substituent effects.

The pure GGA functionals (BP86, BLYP, PBE) consistently yield the smallest HOMO–LUMO energy gaps, typically in the range of 2.4–3.6 eV. The meta-GGA functional TPSS produces slightly larger gaps, ranging from 2.6 to 3.7 eV. A significant increase is observed with the hybrid meta-GGA functional TPSSh, which shows intermediate HOMO–LUMO energy gaps values between 3.3 and 4.4 eV. The hybrid GGA functionals (B3LYP, B3PW91, PBE0, B1LYP) produce the largest gaps, spanning from 3.9 to 5.4 eV. Among the hybrid functionals, a further trend is visible. The global hybrids B3LYP and B3PW91 produce HOMO–LUMO gap values in a similar range, approximately between 3.9 and 5.0 eV. In contrast, the PBE0 and B1LYP functionals typically yield the largest HOMO–LUMO gap values in the data set, approximately between 4.3 and 5.4 eV. This systematic difference can be attributed to the specific amount of exact Hartree–Fock exchange mixed into the functional, with PBE0 using a higher percentage (25%) than B3LYP (20%).

The calculated average energy gap for each molecule is presented in Figure 10a. As shown, the obtained energy gaps fall within the range of 3.298–4.358 eV, with molecule **8** exhibiting the largest gap (4.358 eV), indicating the highest stability. In contrast, molecule **1** presents the smallest gap (3.298 eV), suggesting greater chemical reactivity. This difference of 1.060 eV represents a significant variation in electronic stability, implying that specific structural modifications have a pronounced effect on the electronic properties of the molecules.

An inspection of Figure 9 reveals that the DS12 molecules can be categorized into three stability classes: High stability (Gap >4.2 eV): Molecules **2**, **6**, **7**, **8**, **10**, and **11**; Intermediate stability (3.6–4.2 eV): Molecules **3**, **5**, **9**, and **12**; Low stability (Gap <3.6 eV): Molecules **1** and **4**. The standard deviations in energy gaps (0.769–0.867 eV) indicate consistent functional performance across all molecules, with functional-dependent variations being systematic rather than random.

The electrophilicity index (ω), quantifying electron-accepting capacity, demonstrated remarkable variation (2.393–6.931 eV). Molecule **1**, despite having the lowest stability, shows the highest electrophilicity (6.931 eV), indicating a pronounced electrophilic character. Conversely, molecule **2** presents the lowest electrophilicity (2.393 eV), suggesting a tendency toward nucleophilic behavior. The observed negative correlation between the energy gap and electrophilicity in Figure 10d supports the fundamental DFT principle that molecules with smaller HOMO–LUMO gaps generally display higher chemical reactivity.

Chemical hardness (η), related to resistance to charge transfer, ranged from 1.649 to 2.179 eV. Molecule 8 displayed the maximum hardness (2.179 eV), consistent with its high stability, while molecule **1** showed minimum hardness (1.649 eV). The hardness values follow trends parallel to energy gaps, as expected from their mathematical relationship in DFT.

Electronegativity (χ) varied between 3.167 and 4.634 eV, with molecule **1** exhibiting the highest value (4.634 eV). The exceptionally strong correlation between electronegativity and electrophilicity (*r* = 0.980) indicates that more electronegative molecules tend to be more electrophilic, providing a straightforward design principle for tuning reactivity.

The nine tested functionals showed systematic variations in predicted properties. B1LYP consistently provided the highest energy gaps for most molecules, while PBE0 showed superior performance for specific cases. Hybrid functionals generally predicted larger gaps than pure GGA functionals, consistent with their improved treatment of exchange effects.

The functional-dependent variations (standard deviations of 0.769–0.867 eV in gaps) highlight the importance of functional selection for accurate property prediction. However, the relative trends across molecules remained consistent regardless of functional choice.

For high stability applications, molecules from the high-stability group (**8**, **2**, **7**, **6**, **11**, **10**) with energy gaps >4.2 eV are recommended for applications requiring chemical stability, such as materials science or pharmaceutical development where metabolic stability is crucial.

For electrophilic reactivity, molecules **1** and **4** (ω > 6.0 eV) represent optimal choices for electrophilic reactions or applications requiring electron-accepting character. Their low energy gaps and high electrophilicity make them suitable for charge-transfer applications or as electrophiles in synthetic chemistry.

However, balanced stability-reactivity Profiles molecules **5** and **3** offer intermediate values suitable for applications requiring balanced properties. Their moderate gaps (3.655–3.753 eV) and electrophilicities (3.515–5.362 eV) provide compromise solutions for multiobjective design requirements.

From an applied perspective, diaryl sulfide derivatives have been investigated as potential antiprotozoan inhibitors by Saccoliti and co-workers.^72^ The compound RDS-777 (or 6-(sec-butoxy)-2-((3-chlorophenyl)­thio)­pyrimidin-4-amine) exhibits a geometry similar to that of the DS12 molecules containing chlorine atoms studied in this work. This structural analogy suggests that the present compounds may also exhibit antiprotozoan activity, potentially linked to their pronounced chemical reactivity.

### 3.10 HOMO–LUMO Gap: B3LYP/BP86 vs GFN2-*x*TB

As a comment, the comparative analysis between GFN2-*x*TB and DFT functionals reveals a complex relationship between computational efficiency and predictive accuracy for HOMO–LUMO gap calculations in diaryl sulfide derivatives. The results of the correlational analysis are presented in Table 4. The excellent correlation coefficients (*R* > 0.96) across both functional comparisons indicate that GFN2-*x*TB reliably captures the relative electronic trends and molecular ordering predicted by more computationally intensive DFT methods. This correlation value suggests that the semiempirical method may reproduce the fundamental structure–property relationships governing frontier orbital energetics in these systems.

**Table 4:** Statistical Comparison of GFN2-*x*TB against DFT Functionals

metric	BP86/GFN2-*x*TB	B3LYP/GFN2-*x*TB
*R*	0.962	0.967
*R* ^2^	0.925	0.935
MAE (eV)	0.315	1.821
RMSE (eV)	0.347	1.826
relative error (%)	10.45	39.49

However, the substantial quantitative discrepancies, particularly against the hybrid functional B3LYP, highlight fundamental methodological differences. The systematic underestimation of energy gaps by GFN2-*x*TB appears directly related to the absence of exact exchange in its methodology. The significantly better agreement with BP86 (10.45% average error) compared to B3LYP (39.49% average error) suggests that GFN2-*x*TB’s parametrization aligns more closely with GGA-type functionals than hybrid approaches. This methodological alignment explains the consistent scaling behavior observed across the entire molecular series.

The practical implications of these findings are significant for computational screening workflows. For applications requiring absolute quantitative accuracy, particularly those benchmarking against experimental values typically closer to hybrid functional predictions, traditional DFT remains necessary. However, for high-throughput virtual screening where relative compound ranking drives decision-making, GFN2-*x*TB offers an exceptional balance between computational cost and predictive capability.

### 3.11 Effects on Dispersion Corrections Interaction on Molecular Properties

Table 5 presents a comprehensive comparison of four electronic structure methods: BP86, BP86/D3BJ, B3LYP, and B3LYP/D3BJ for calculating key molecular properties across DS12 molecules. For each molecule, the results are presented in four rows per column: dipole moment (Debye), ionization potential (IP, eV), electron affinity (EA, eV), and HOMO–LUMO energy gap (eV).

**Table 5:** Comparison of Dipole Moment (in Debye, First Line), Ionization Potential (in eV, Second Line), Electron Affinity (in eV, Third Line), and HOMO–LUMO Energy Gap (in eV, Fourth Line)

molecule	BP86	BP86/D3BJ	B3LYP	B3LYP/D3BJ
**1**	4.864	4.825	5.033	4.994
	7.808	7.786	7.893	7.870
	1.452	1.463	1.216	1.224
	2.428	2.404	3.915	3.894
**2**	5.886	5.761	5.595	5.491
	6.457	6.449	6.453	6.446
	–0.095	0.090	0.228	0.294
	3.460	3.442	4.960	4.952
**3**	3.196	3.167	2.913	2.903
	7.821	7.797	7.882	7.864
	1.300	1.330	0.982	1.006
	2.915	2.886	4.366	4.338
**4**	4.566	4.452	4.597	4.522
	7.662	7.646	7.799	7.792
	1.361	1.363	1.008	0.973
	2.537	2.513	4.096	4.112
**5**	5.691	5.568	5.577	5.515
	6.741	6.721	6.802	6.781
	0.495	–0.480	0.099	–0.084
	2.857	2.837	4.240	4.226
**6**	0.823	0.759	0.778	0.756
	7.357	7.358	7.360	7.356
	0.001	–0.0003	–0.400	0.404
	3.364	3.347	4.886	4.875
**7**	2.465	2.500	2.554	2.591
	7.148	7.141	7.173	7.165
	–0.156	0.159	–0.501	0.511
	3.462	3.449	4.968	4.950
**8**	3.867	3.809	3.880	3.813
	7.649	7.645	7.650	7.637
	0.515	0.503	0.220	0.206
	3.456	3.461	5.018	5.021
**9**	1.303	1.309	1.244	1.245
	7.359	7.358	7.391	7.395
	0.229	0.227	–0.138	0.148
	3.305	3.287	4.830	4.814
**10**	2.001	2.058	2.553	2.385
	7.179	7.176	7.194	7.187
	–0.007	0.008	–0.414	0.414
	3.348	3.317	4.877	4.838
**11**	1.849	1.884	1.972	1.992
	6.994	6.989	6.987	6.979
	–0.146	0.143	–0.562	0.562
	3.381	3.361	4.883	4.858
**12**	3.358	3.289	3.274	3.216
	7.171	7.168	7.200	7.193
	0.093	0.094	–0.116	0.145
	3.302	2.886	4.848	4.339

The calculated dipole moments show moderate sensitivity to both the choice of functional and the inclusion of dispersion corrections. The inclusion of the D3BJ correction generally induces minor changes (typically <0.2 D for most molecules), suggesting that dispersion effects have a small but non-negligible influence on electron density distribution. For instance, in molecule **2**, the dipole moment decreases from 5.886 D (BP86) to 5.761 D (BP86/D3BJ), while in molecule **10**, it increases from 2.001 to 2.058 D with the same correction.

The vertical ionization potentials demonstrate remarkable consistency across all computational methods. For any given molecule, the variation between the four computed IPs is consistently less than 0.1 eV, and often less than 0.05 eV. The negligible effect of the D3BJ correction confirms that dispersion interactions contribute minimally to the energy difference between a neutral molecule and its cation, as expected for a property dominated by the removal of a single, localized electron. In contrast to the ionization potentials, the electron affinities exhibit pronounced functional dependence and high sensitivity to dispersion corrections. Strictly speaking, the inclusion of the D3BJ correction exerts a dramatic and often decisive influence on both the sign and magnitude of the EA, particularly for the B3LYP functional. For molecules **6, 7, 9, 10, 11, and 12**, the uncorrected B3LYP functional predicts negative EAs (indicating endothermic electron attachment), whereas B3LYP/D3BJ predicts positive, physically reasonable values. The BP86 functional shows similar trends, with the correction shifting EAs toward more positive values, though the effect is sometimes less pronounced (e.g., molecule **5**). These results highlight that stabilizing dispersion interactions in the more diffuse anion are crucial for obtaining correct qualitative and quantitative descriptions of electron affinity. Neglecting these interactions can lead to qualitatively incorrect predictions regarding anion stability.

The HOMO–LUMO gap also demonstrates systematic variation between the two families of functionals. As theoretically anticipated, the hybrid B3LYP functional yields significantly larger gaps (by approximately 1.5–1.7 eV on average) than the pure GGA BP86 functional. The effect of the D3BJ correction on the gap is generally minimal, typically altering values by less than 0.03 eV. However, one notable anomaly occurs for molecule **12** with the BP86/D3BJ method, where the reported gap (2.886 eV) is 0.416 eV lower than the uncorrected BP86 value. This outlier warrants further investigation, as it may indicate a significant conformational change or electronic structure effect induced specifically by dispersion interactions for this system.

### 3.12 Sensitivity of High-Level Energies to Optimized Geometries

To conclude this analysis, we conducted a geometry sensitivity test for four representative molecules (**1**, **3**, **8**, and **10**) from the DS12 set. The test compares DLPNO–CCSD­(T)/cc-pVTZ single-point energies and dipole moments calculated on geometries optimized with three different functionals: B3LYP, PBE0, and TPSSh.

The DLPNO–CCSD­(T1) total energies obtained from B3LYP and TPSSh optimized geometries are remarkably consistent. The maximum relative energy difference (Δ*E*_rel_) is only 0.5 kcal/mol (for molecule **1**), which is well within chemical accuracy thresholds. For molecules **3** and **8**, the differences are <0.3 kcal/mol. This indicates that high-level energetics are not sensitive to the choice between these two functionals for geometry optimization. Geometries optimized with PBE0 lead to systematically lower (more negative) DLPNO–CCSD­(T) energies compared to B3LYP, with differences of approximately 5 kcal/mol.

The DLPNO–CCSD­(T) dipole moments exhibit a moderate dependence on the input geometry, yielding relative differences (Δμ_rel_) of −0.01 to 0.7 D. Figure S9 in the Supporting Information illustrates these variations. This sensitivity test demonstrates that while the PBE0 functional induces a consistent energy shift, the B3LYP and TPSSh functionals produce geometries that yield virtually equivalent high-level single-point energies.

## 4 Conclusions

A systematic investigation of 12 strategically designed diaryl sulfide (DS12) derivatives was conducted using density functional theory (DFT), revealing clear structure–property relationships and providing practical guidance for computational method selection. The study demonstrates that subtle structural modifications can induce significant variations in electronic properties. The molecular set, which encompasses a broad spectrum of substituent effects including strong electron-withdrawing groups (e.g., NO_2_, CF_3_, halogens), electron-donating groups (e.g., NH_2_, OCH_3_), and neutral moieties in both symmetric and asymmetric patterns, allowed for a comprehensive analysis of electronic tuning.

Key trends in electronic properties were identified. HOMO–LUMO energy gaps varied substantially from 3.298 to 4.358 eV, with molecule **8** emerging as the most stable and molecule **1** exhibiting the highest reactivity. A pronounced inverse correlation was observed between the energy gap and the electrophilicity index (ω), which itself spanned a range of 4.538 eV, highlighting the direct link between stability and chemical reactivity. Molecular polarity, as measured by dipole moments, also showed considerable variation. Molecules **2** and **5** were the most polar, with average dipole moments of approximately 5.7 D, while molecule **6** displayed remarkably low polarity at about 1.0 D. The DFT ensemble average for dipole moments correlated excellently with high-level DLPNO–CCSD­(T) reference values, yielding a correlation coefficient of *R* = 0.983.

The performance of various computational methods was benchmarked to offer practical recommendations. For the accurate prediction of dipole moments, the hybrid functionals **B1LYP** and **B3LYP** proved most reliable, exhibiting the lowest Mean Absolute Errors (MAE ≈ 0.25 D). For calculating vertical transition energies (S_0_ → S_1_ and S_0_ → T_1_), the hybrid-meta GGA functional **TPSSh** demonstrated superior accuracy with MAEs of 0.10 and 0.06 eV, respectively, closely followed by the hybrid GGA functional **B3PW91**. These results indicate a performance hierarchy that mirrors Jacob’s Ladder classification. This interpretation is corroborated by the observation that pure generalized gradient approximation (GGA) functionals consistently and significantly underestimate excitation energies relative to the high-level STEOM-DLPNO–CCSD reference, yielding large mean absolute errors (0.62–0.63 eV). Therefore, the hierarchical principle of Jacob’s Ladder remains a valid and useful framework for guiding functional selection in time-dependent DFT calculations of diaryl sulfide systems.

Geometrically, DFT methods predicted remarkably consistent C–S–C bond angles across all molecules, clustering around 104.0°. The semiempirical GFN2-*x*TB method provided good qualitative structural insights at a fraction of the computational cost but exhibited systematic quantitative deviations, particularly in shortening bonds to heteroatoms and misrepresenting torsional angles of flexible groups like nitro.

The role of dispersion corrections was critically assessed. The inclusion of the D3BJ correction was found to be essential for obtaining physically meaningful electron affinities, especially when using hybrid functionals, as it accounts for stabilizing interactions in the more diffuse anion. Structurally, D3BJ induced predictable contractions, most notably in C–S bond lengths, but had a minimal effect on the accuracy of vertical excitation energy calculations.

Based on the calculated properties, guidance for molecular design is provided. For applications requiring high kinetic stability, such as in materials science, molecules **8**, **2**, **7**, **6**, **11**, and **10** (with HOMO–LUMO gaps >4.2 eV) are recommended. Conversely, for applications demanding high electrophilic reactivity, such as in charge-transfer processes or as synthetic intermediates, molecules **1** and **4** (with ω > 6.0 eV) are identified as optimal choices. Molecules like **5** and **3**, which offer intermediate values, present suitable candidates for applications requiring a balanced stability-reactivity profile.

This study establishes a comprehensive computational benchmark within the gas-phase approximation. It is acknowledged that future work should incorporate solvent effects for greater practical relevance, seek experimental validation of the predicted properties, and explore dynamic processes and kinetic parameters. Nevertheless, the findings provide a solid foundation for the rational, property-targeted design of diaryl sulfide-based systems for applications in organic electronics, catalysis, and medicinal chemistry.

## Supplementary Material





## Notes

Global reactivity descriptors of the molecules 1–12 (CSV)
Optimized geometries, HOMO–LUMO energy gaps, and global reactivity descriptor and dipole moment values (PDF)
