# Supplementary material for: Designing Diaryl Sulfide Electronic Properties: A Comprehensive DFT Guide to HOMO–LUMO Gaps and Reactivity Descriptors
Source: ACS Omega. 2026 May 1;11(18):26328–44. doi: 10.1021/acsomega.5c11432 (PMC13177040; doi:10.1021/acsomega.5c11432)
Supplement: Supplementary file 2 [file ao5c11432_si_002.pdf]

Designing Diaryl Sulfide Electronic Properties: A Comprehensive DFT Guide to HOMO-LUMO Gaps and Reactivity Descriptors Supporting Information

Ramon S. da Silva*

Departamento de Física, Universidade Federal de Juiz de Fora,

Juiz de Fora, 36036-330, Minas Gerais, Brazil

* ramon.sousa@ufjf.br

The statistical parameters used to evaluate the performance of the employed functionals are defined as follows. For a set of n data points, where y_i represents the reference value and $\hat{y}_i$ represents the calculated value, the metrics are defined as:

- Mean Absolute Error (MAE):

$$\text{MAE} = \frac{1}{n}\sum_{i=1}^{n} |y_i - \hat{y}_i| \tag{S1}$$

- Root Mean Square Error (RMSE):

$$\text{RMSE} = \sqrt{\frac{1}{n}\sum_{i=1}^{n}(y_i - \hat{y}_i)^2} \tag{S2}$$

- Standard Deviation (SD) of the errors:

$$\text{SD} = \sqrt{\frac{1}{n-1}\sum_{i=1}^{n}\left((y_i - \hat{y}_i) - \bar{\epsilon}\right)^2} \tag{S3}$$

where $\bar{\epsilon} = \frac{1}{n}\sum_{i=1}^{n}(y_i - \hat{y}_i)$ is the mean error.

TABLE S1. Selected diaryl sulfide species

Compound	names
1	2-[(2,4-Dichlorophenyl)thio]nitrobenzene
2	4-((4-Chlorophenyl)thio)-N,N-dimethylaniline
3	3-Bromo-4-[(2,4-dichlorophenyl)thio]benzaldehyde
4	5-Bromo-2-[(2,4-dichlorophenyl)thio]benzaldehyde
5	5-Chloro-4-[(4-chlorophenyl)sulfanyl]aniline
6	(4-Fluorophenyl)(phenyl)sulfane
7	(3-methoxyphenyl) phenyl sulfane
8	4-(Trifluoromethyl)phenyl phenyl sulfide
9	(4-Chlorophenyl)(4-fluorophenyl)sulfane
10	(4-fluorophenyl)(p-tolyl)sulfane
11	4-Tolyl phenyl sulfide
12	(4-chlorophenyl)(p-tolyl)sulfane

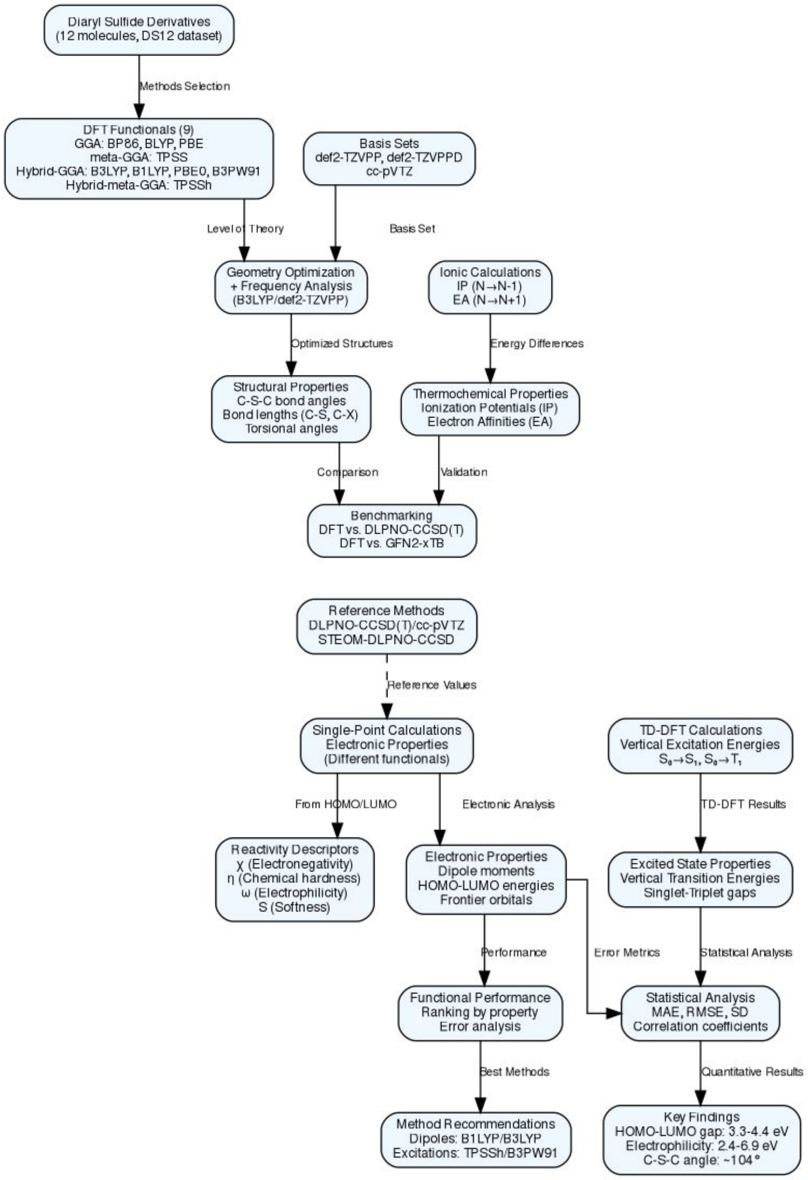

FIG. S1. Computational workflow for the DFT study of diaryl sulfide derivatives. The protocol encompasses: (1) definition of the DS12 molecular dataset; (2) selection of computational methods including 9 DFT functionals and high-level reference calculations; (3) geometry optimizations and property calculations; (4) comprehensive analysis of structural, electronic, and thermochemical properties.

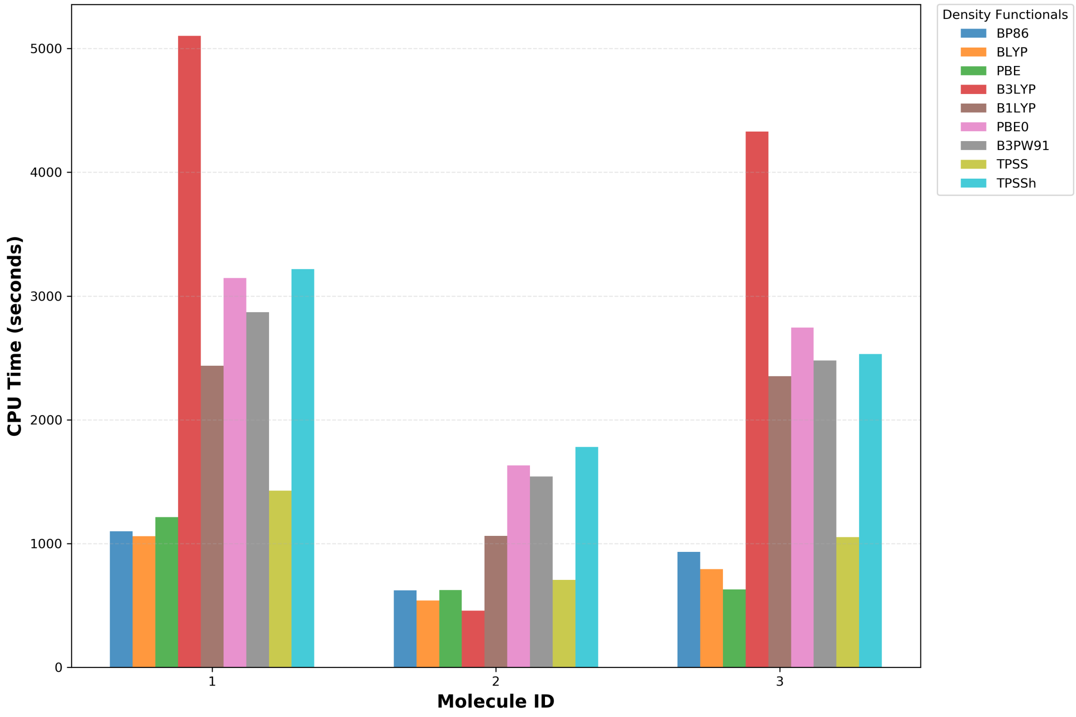

FIG. S2. Computational cost comparison: CPU time versus molecule ID for different density functionals.

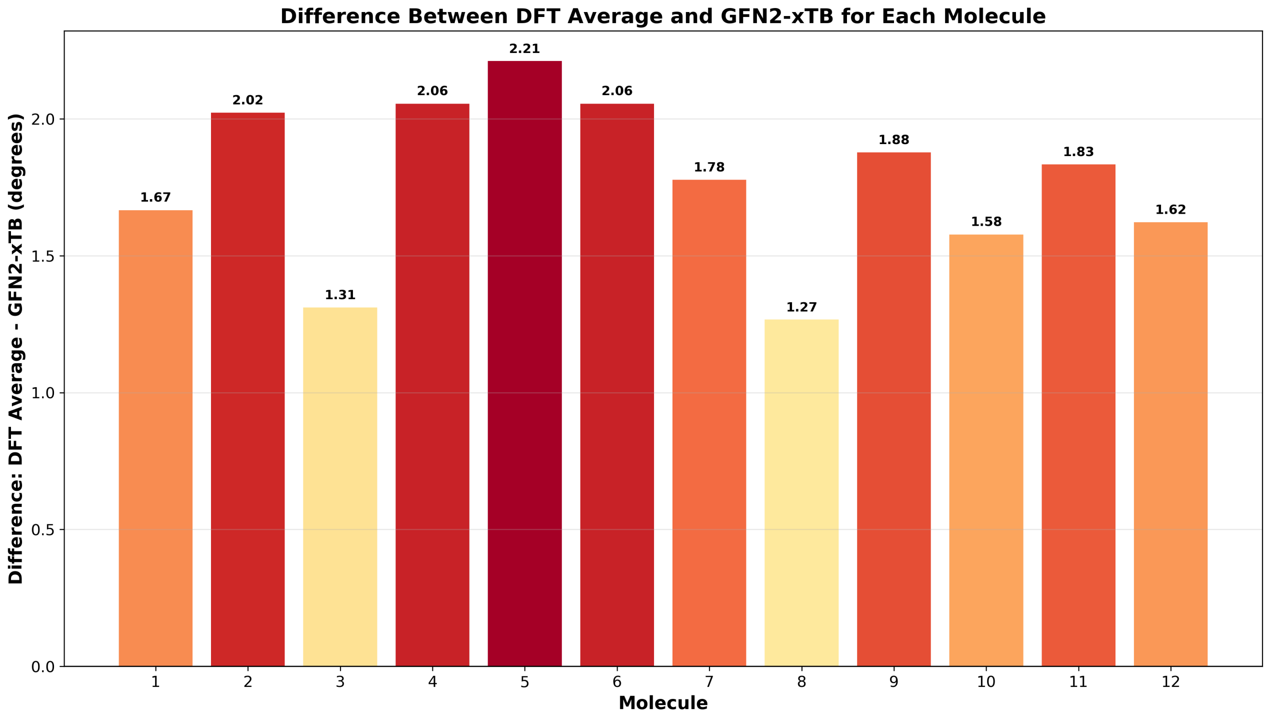

FIG. S3. Systematic Deviation Analysis: DFT Average Minus GFN2-xTB Bond Angles

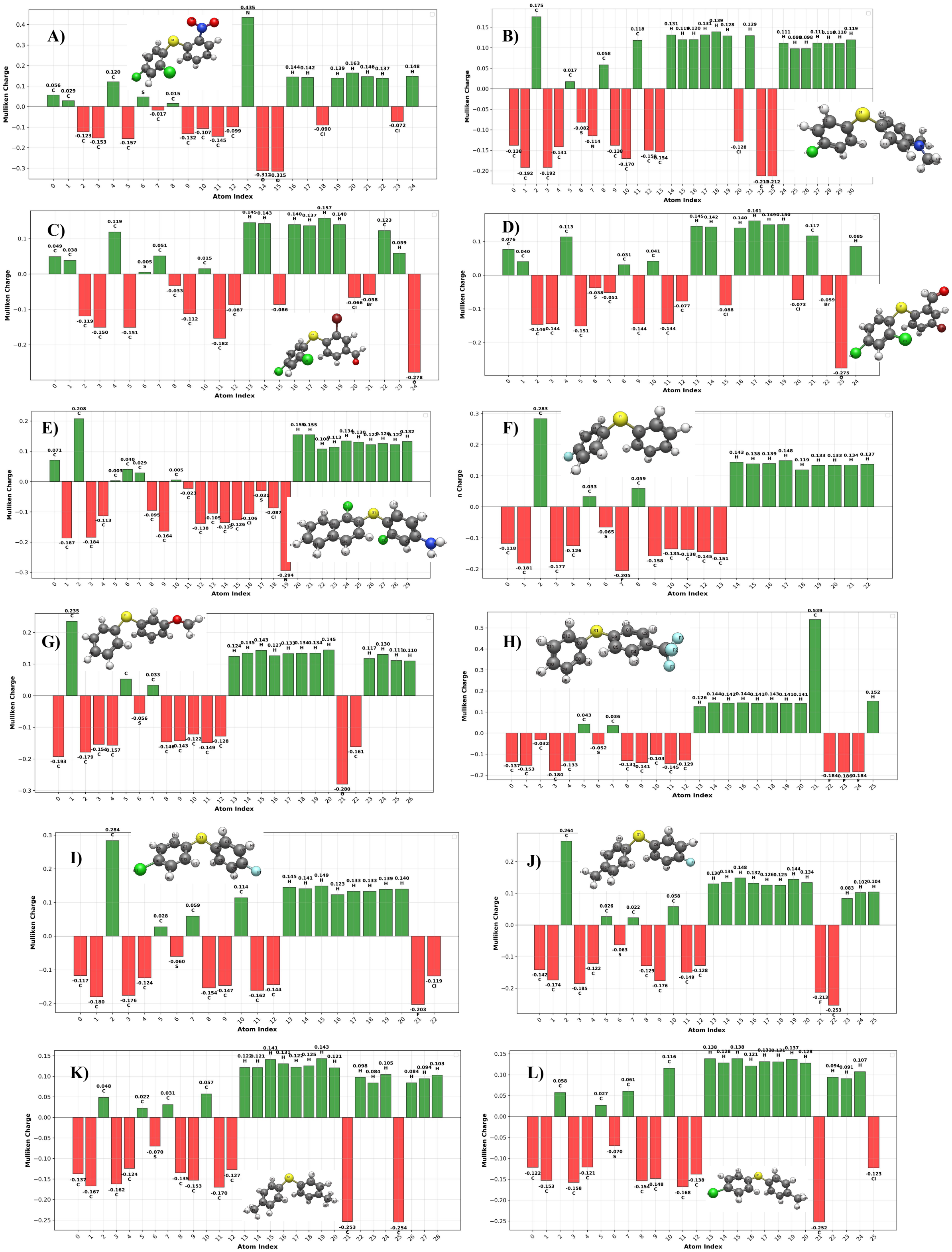

FIG. S4. Mulliken charge distribution vs atom index for DS12 molecules: A) **1**, B) **2**, C) **3**, D) **4**, E) **5**, F) **6**, G) **7**, H) **8**, I) **9**, J) **10**, K) **11**, L) **12**. The corresponding names are given in Table S1.

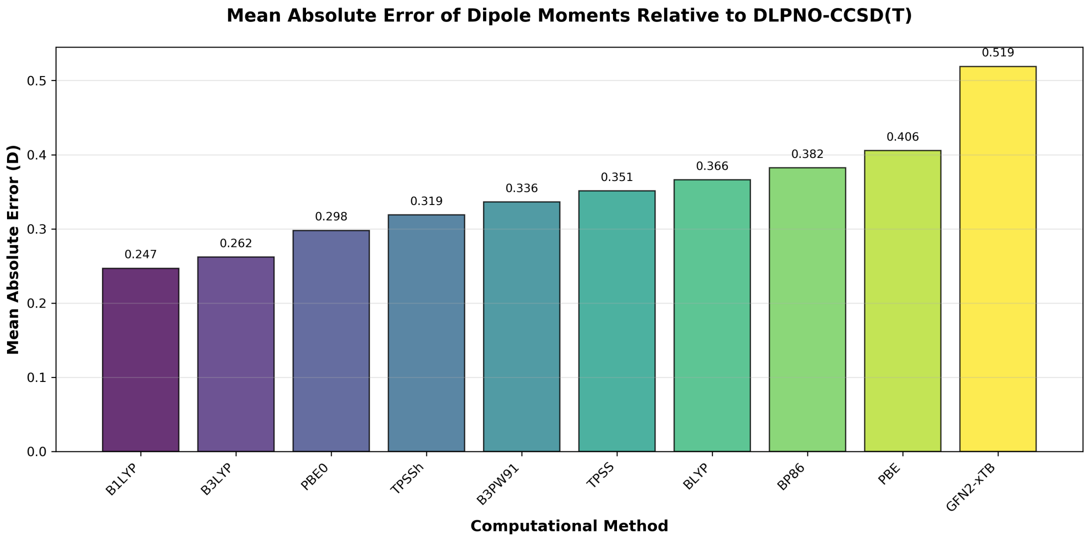

FIG. S5. Performance ranking of computational methods for dipole moment prediction. Bar chart displays Mean Absolute Error (MAE) relative to DLPNO-CCSD(T) reference, with methods sorted by increasing error. DFT functionals B3LYP, PBE0, and B1LYP demonstrate superior performance with MAE values below 0.3 D.

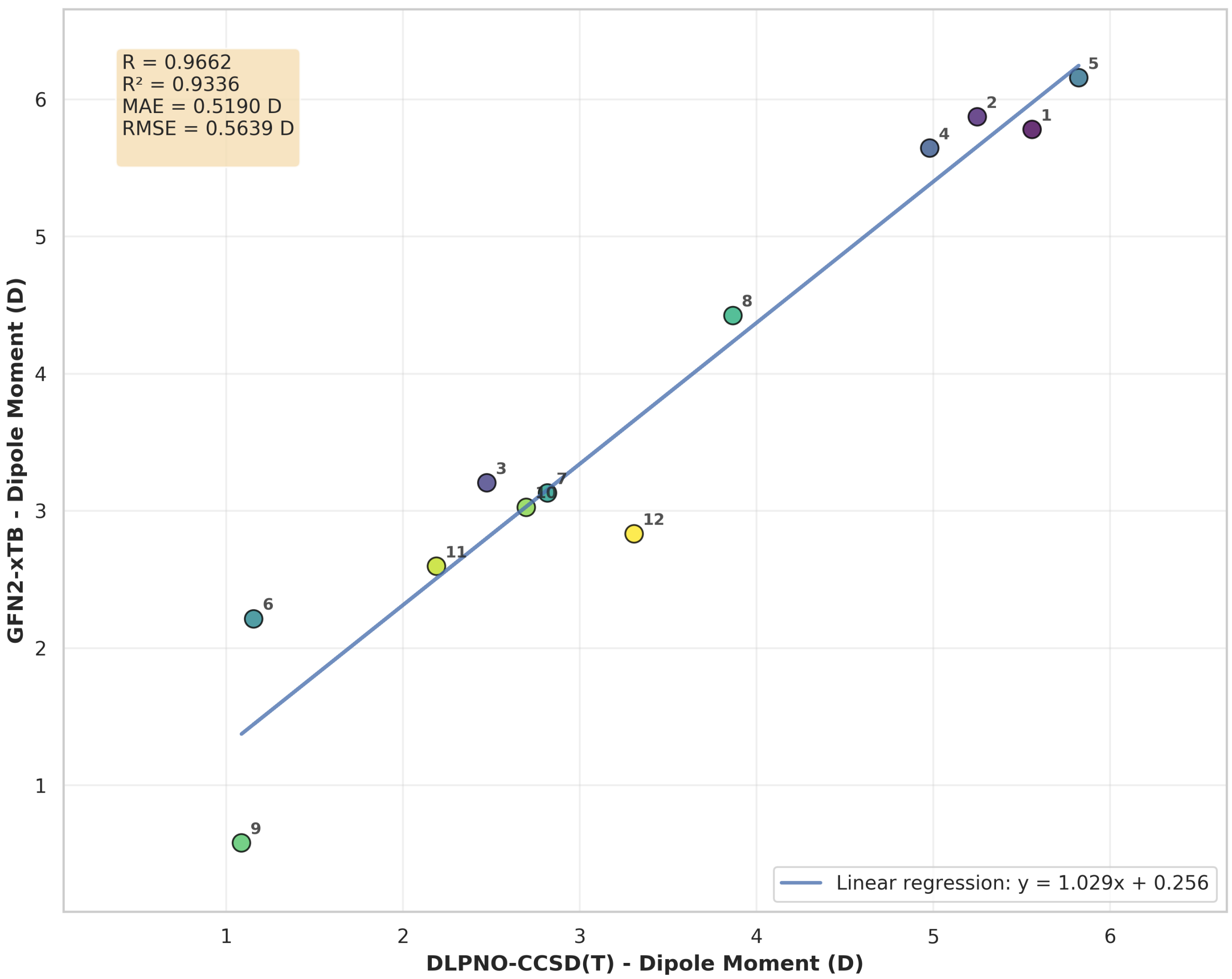

FIG. S6. Correlation between DLPNO-CCSD(T) and GFN2-xTB dipole moment predictions for 12 molecular systems. The blue line represents the linear regression fit, with each point labeled by molecule ID.

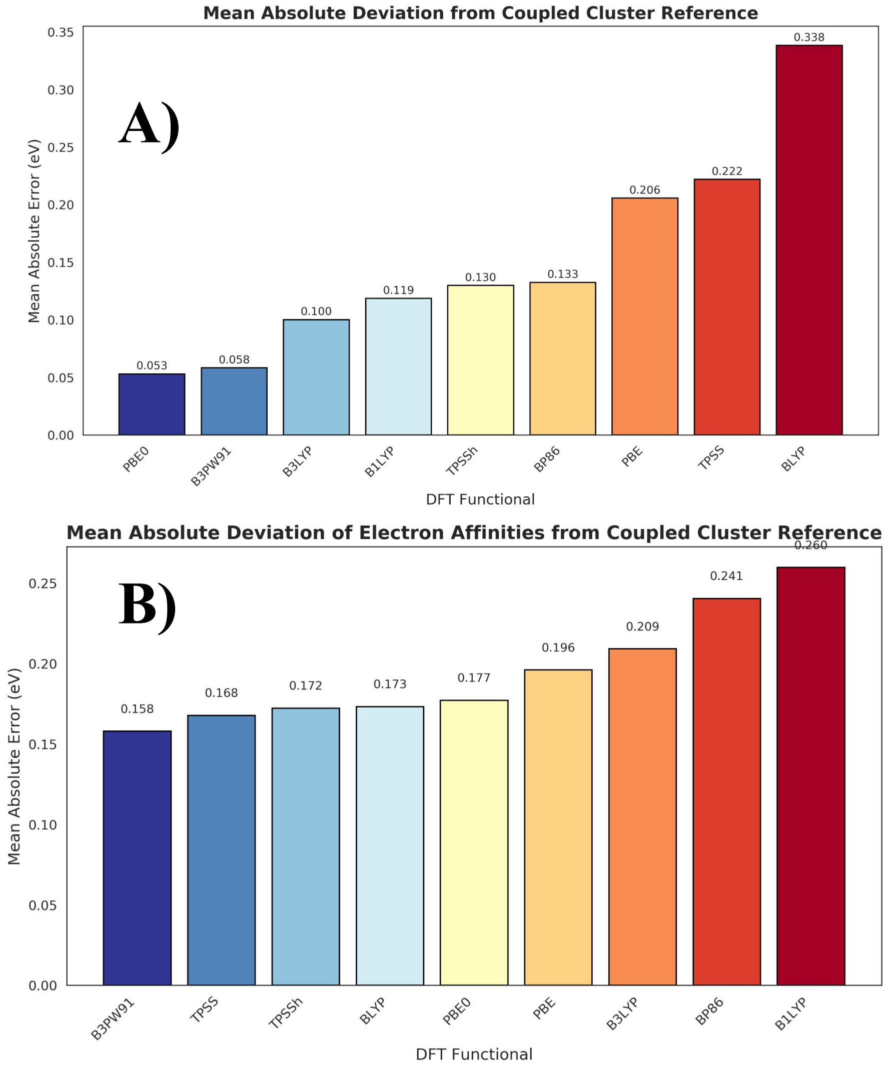

FIG. S7. Mean absolute error (MAE) of DFT functionals relative to the coupled-cluster reference: A) Ionization potential (IP) and B) Electron affinity (EA). The MAE (in eV) is calculated across all twelve molecules for each functional. Functionals are ordered by increasing MAE, providing a direct visual assessment of their overall accuracy for IP and EA in this test set.

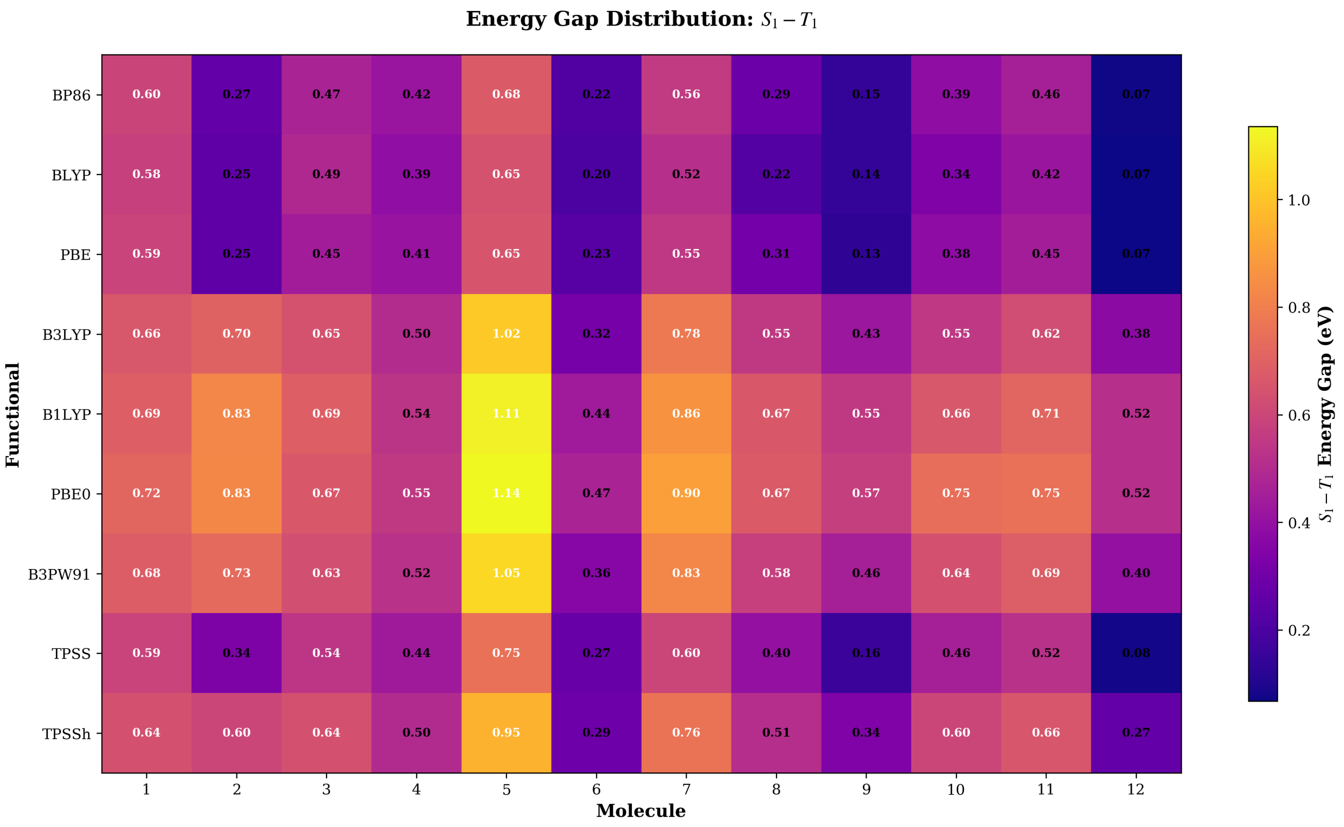

FIG. S8. Simplified representation of density functional dependence on computed S_1–T_1 energy gaps across twelve molecular systems

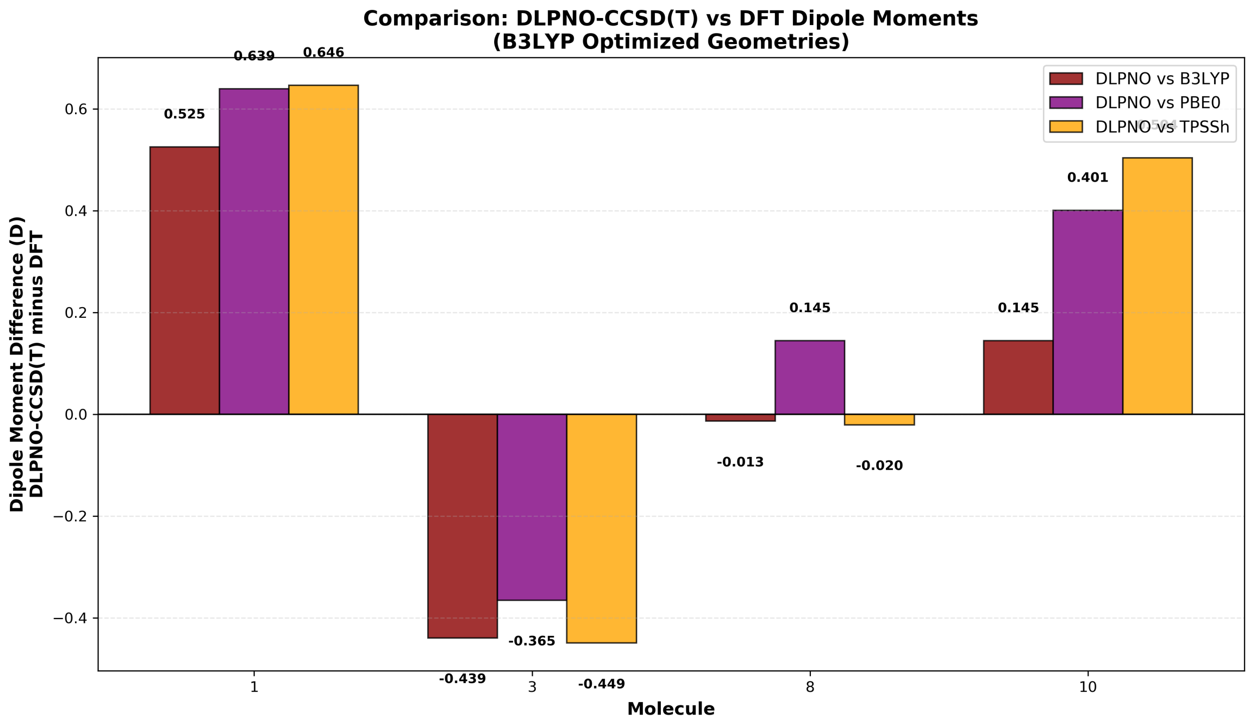

FIG. S9. Differences Between DLPNO-CCSD(T) and DFT Dipole Moments.

TABLE S2. Dipole moment (D): DFT/def2-TZVPP results versus DLPNO-CCSD(T)/cc-pVTZ reference.

Molecule	BP86	BLYP	PBE	B3LYP	B1LYP	PBE0	B3PW91	TPSS	TPSSh	DLPNO-CCSD(T)	B3LYP/D3
**1**	4.864	4.964	4.808	5.033	5.049	4.919	4.959	4.873	4.912	5.558	4.994
**2**	5.886	5.853	5.847	5.595	5.536	5.556	5.640	5.862	5.732	5.248	5.491
**3**	3.196	3.199	3.213	2.913	2.846	2.839	2.895	3.061	2.923	2.474	2.903
**4**	4.566	4.579	4.527	4.597	4.602	4.559	4.585	4.575	4.582	4.979	4.522
**5**	5.691	5.645	5.652	5.577	5.551	5.575	5.631	5.671	5.650	5.822	5.515
**6**	0.823	0.923	0.826	0.778	0.807	0.783	0.774	0.909	0.789	1.155	0.756
**7**	2.465	2.470	2.420	2.554	2.578	2.558	2.552	2.473	2.533	2.817	2.591
**8**	3.867	4.046	3.803	3.880	3.859	3.722	3.781	3.837	3.887	3.866	3.813
**9**	1.303	1.159	1.331	1.244	1.225	1.280	1.293	1.311	1.321	1.086	1.245
**10**	2.001	2.030	1.959	2.553	2.527	2.297	1.809	2.128	2.194	2.697	2.385
**11**	1.849	1.821	1.816	1.972	1.993	2.008	1.985	1.863	1.926	2.189	1.992
**12**	3.358	3.332	3.318	3.274	3.260	3.261	3.299	3.343	3.314	3.307	3.216

TABLE S3. Ionization Potentials (eV) for the DS12 test set. Results obtained with various density functional theory (DFT) functionals are compared to high-level reference values from the DLPNO-CCSD(T) method (denoted as CC).

Molecule	BP86	BLYP	PBE	B3LYP	B1LYP	PBE0	B3PW91	TPSS	TPSSh	CC
1	7.808	7.589	7.712	7.893	7.865	7.986	7.995	7.717	7.838	7.978
2	6.457	6.228	6.365	6.453	6.406	6.550	6.565	6.323	6.414	6.396
3	7.821	7.593	7.722	7.882	7.858	7.994	8.000	7.732	7.842	7.9473
4	7.662	7.442	7.563	7.799	7.784	7.901	7.907	7.591	7.741	7.898
5	6.741	6.514	6.646	6.802	6.787	6.911	6.918	6.629	6.747	6.988
6	7.357	7.138	7.267	7.360	7.317	7.448	7.462	7.235	7.322	7.422
7	7.148	6.914	7.064	7.173	7.142	7.286	7.287	7.015	7.129	7.261
8	7.649	7.425	7.561	7.650	7.600	7.732	7.739	7.528	7.604	7.831
9	7.359	7.140	7.265	7.391	7.370	7.493	7.504	7.264	7.358	7.491
10	7.179	6.956	7.088	7.194	7.150	7.278	7.293	7.066	7.152	7.273
11	6.994	6.771	6.910	6.987	6.949	7.087	7.100	6.884	6.966	7.109
12	7.171	6.948	7.086	7.200	7.159	7.292	7.305	7.069	7.163	7.122

TABLE S4. Dipole Moments (D) for cation especies calculated with different DFT functionals

Molecule	BP86	BLYP	PBE	B3LYP	B1LYP	PBE0	B3PW91	TPSS	TPSSh
1	4.82954	4.98960	4.75872	5.21608	5.27982	5.02972	5.06106	4.87587	4.97471
2	9.78932	9.96692	9.63053	11.20126	11.72373	11.07475	10.98276	10.09911	10.69524
3	2.88161	3.00504	2.84939	3.42662	3.26096	3.27226	3.35400	2.95735	3.37905
4	5.13343	5.19889	5.08587	4.91884	4.63172	4.73622	4.93321	5.18799	5.20091
5	6.95745	6.90195	6.98747	7.04752	7.09114	7.18761	7.07904	7.01246	7.05649
6	2.43316	2.59336	2.40338	2.67981	2.72214	2.54339	2.55747	2.51325	2.55935
7	2.16293	2.16187	2.13995	2.05171	2.06006	2.04188	2.04677	2.26475	2.16386
8	9.73780	9.98512	9.67730	10.24938	10.36331	10.04527	10.04801	9.85309	9.97294
9	1.40378	1.41578	1.36757	1.67709	1.75208	1.72196	1.65872	1.46284	1.59525
10	3.13825	3.23691	3.10848	3.47106	3.58678	3.40479	3.36740	3.11952	3.21050
11	1.16541	1.09951	1.16435	0.95325	0.90591	0.95804	0.99448	1.14027	1.05975
12	3.74017	3.74932	3.67625	4.05566	4.16914	4.03893	4.00637	3.77334	3.89102

TABLE S5. Electron affinities (eV) for the DS12 test set. Results obtained with various density functional theory (DFT) functionals are compared to high-level reference values from the DLPNO-CCSD(T) method (denoted as CC).

Molécula	BP86	BLYP	PBE	B3LYP	B1LYP	PBE0	B3PW91	TPSS	TPSSh	CC
1	1.452	1.241	1.334	1.216	1.142	1.230	1.288	1.278	1.235	1.893
2	-0.095	-0.334	-0.197	3.228	-4.330	-0.377	-0.339	-0.309	-0.384	1.136
3	1.300	1.064	1.198	0.982	0.888	1.035	1.088	1.116	1.049	1.155
4	1.361	1.112	1.256	1.008	0.907	1.064	1.121	1.166	1.088	1.359
5	0.495	0.243	0.385	0.099	0.246	0.403	0.452	0.438	0.394	0.295
6	0.001	-0.243	-0.101	-0.400	-0.560	-0.359	-0.294	-0.231	-0.327	-0.337
7	-0.156	-0.368	-0.257	-0.501	-0.610	-0.469	-0.406	-0.375	-0.460	-0.563
8	0.515	0.297	0.412	0.220	0.129	0.262	0.318	0.319	0.266 0.	660
9	0.229	-0.014	0.125	-0.138	-0.209	-0.082	-0.030	0.013	-0.072	0.152
10	-0.007	-0.251	-0.105	-0.414	-0.532	-0.373	-0.309	-0.237	-0.336	-0.310
11	-0.146	-0.404	-0.238	-0.562	-0.675	-0.506	-0.446	-0.372	-0.470	-0.520
12	0.093	-0.162	-0.005	-0.116	-0.173	-0.195	-0.146	-0.118	-0.199	-0.120

TABLE S6. Vertical excitation, VE, energies (in eV) for the DS12 test set. Results obtained with TDDFT/TDA approach are compared to high-level reference values from the STEOM-DLPNO-CCSD method (denoted as CC).

Molecule	VE	BP86	BLYP	PBE	B3LYP	B1LYP	PBE0	B3PW91	TPSS	TPSSh	CC	B3LYP/D3	BP86/D3
**1**	$\Delta E^{S_0\to S_1}$	2.896	2.837	2.907	3.431	3.571	3.631	3.483	3.010	3.303	3.660	3.386	2.850
	$\Delta E^{S_0\to T_1}$	2.300	2.258	2.317	2.768	2.881	2.913	2.799	2.421	2.662	3.097	2.763	2.279
**2**	$\Delta E^{S_0\to S_1}$	3.571	3.579	3.560	4.297	4.447	4.437	4.314	3.767	4.134	4.133	4.279	3.554
	$\Delta E^{S_0\to T_1}$	3.305	3.325	3.307	3.595	3.621	3.607	3.587	3.431	3.536	3.239	3.602	3.295
**3**	$\Delta E^{S_0\to S_1}$	3.158	3.182	3.135	3.680	3.783	3.746	3.663	3.323	3.583	3.650	3.675	3.140
	$\Delta E^{S_0\to T_1}$	2.687	2.689	2.689	3.035	3.093	3.079	3.029	2.784	2.943	2.796	3.026	2.669
**4**	$\Delta E^{S_0\to S_1}$	2.808	2.804	2.791	3.379	3.507	3.472	3.379	2.973	3.255	3.541	3.336	2.753
	$\Delta E^{S_0\to T_1}$	2.390	2.412	2.379	2.875	2.970	2.919	2.854	2.537	2.756	2.945	2.867	2.365
**5**	$\Delta E^{S_0\to S_1}$	3.243	3.241	3.233	3.829	3.950	3.946	3.839	3.393	3.686	3.664	3.806	3.178
	$\Delta E^{S_0\to T_1}$	2.567	2.594	2.579	2.811	2.836	2.811	2.787	2.640	2733	2.502	2.815	2.566
**6**	$\Delta E^{S_0\to S_1}$	3.463	3.473	3.463	4.066	4.212	4.219	4.089	3.654	3.933	3.783	4.068	3.437
	$\Delta E^{S_0\to T_1}$	3.246	3.269	3.230	3.750	3.772	3.749	3.726	3.379	3.642	3.435	3.747	3.241
**7**	$\Delta E^{S_0\to S_1}$	3.791	3.786	3.778	4.361	4.486	4.472	4.373	3.935	4.235	3.984	4.342	3.778
	$\Delta E^{S_0\to T_1}$	3.233	3.271	3.230	3.577	3.622	3.571	3.547	3.336	3.474	3.311	3.561	3.220
**8**	$\Delta E^{S_0\to S_1}$	3.559	3.529	3.568	4.144	4.289	4.281	4.156	3.762	4.026	4.187	4.148	3.552
	$\Delta E^{S_0\to T_1}$	3.273	3.305	3.262	3.590	3.621	3.607	3.577	3.364	3.514	3.249	3.592	3.284
**9**	$\Delta E^{S_0\to S_1}$	3.368	3.386	3.348	4.041	4.193	4.188	4.056	3.557	3.887	4.344	4.044	3.355
	$\Delta E^{S_0\to T_1}$	3.223	3.244	3.216	3.616	3.638	3.613	3.597	3.396	3.548	3.259	3.616	3.207
**10**	$\Delta E^{S_0\to S_1}$	3.547	3.552	3.529	4.143	4.309	4.283	4.168	3.736	4.031	4.095	4.137	3.529
	$\Delta E^{S_0\to T_1}$	3.162	3.212	3.147	3.592	3.645	3.536	3.527	3.280	3.432	3.305	3.531	3.128
**11**	$\Delta E^{S_0\to S_1}$	3.615	3.631	3.600	4.175	4.309	4.286	4.190	3.792	4.071	4.221	4.152	3.593
	$\Delta E^{S_0\to T_1}$	3.158	3.211	3.150	3.550	3.596	3.534	3.505	3.269	3.413	3.271	3.525	3.140
**12**	$\Delta E^{S_0\to S_1}$	3.326	3.329	3.311	4.001	4.157	4.138	4.003	3.504	3.831	4.265	4.008	3.334
	$\Delta E^{S_0\to T_1}$	3.253	3.261	3.240	3.621	3.641	3.621	3.603	3.427	3.564	3.260	3.622	3.262

TABLE S7. Primary orbital transition configurations for the lowest singlet (S_1) and triplet (T_1) excited states of compounds **1–12** from TDDFT calculations. Only contributions $\geq 2\%$ are listed. H = HOMO, L = LUMO.

Molecule		Main Transition Configuration
1	$S_0 \rightarrow S_1$	H → L 96%
	$S_0 \rightarrow T_1$	H → L 92%, H - 3 → L 3%
2	$S_0 \rightarrow S_1$	H -1 → L 92%
	$S_0 \rightarrow T_1$	H → L 94%, H - 2 → L 2%
3	$S_0 \rightarrow S_1$	H -1 → L 90%, H -2 → L 3%
	$S_0 \rightarrow T_1$	H → L 90%, H - 3 → L 3%
4	$S_0 \rightarrow S_1$	H → L 69%, H -1 → L 24%
	$S_0 \rightarrow T_1$	H → L 67%, H - 1 → L 13%, H - 2 → L 12%
5	$S_0 \rightarrow S_1$	H → L 98%
	$S_0 \rightarrow T_1$	H → L 79%, H - 1 → L 10%, H - 2 → L 5%
6	$S_0 \rightarrow S_1$	H → L 60%, H → L + 1 39%
	$S_0 \rightarrow T_1$	H → L 90%, H - 1 → L 5%, H - 2 → L 2%
7	$S_0 \rightarrow S_1$	H → L 94%
	$S_0 \rightarrow T_1$	H → L 79%, H → L + 1 5%, H - 2 → L 2%
8	$S_0 \rightarrow S_1$	H → L 93%, H → L + 1 4%
	$S_0 \rightarrow T_1$	H → L 15%, H → L + 1 45%, H → L + 2 27%
9	$S_0 \rightarrow S_1$	H → L 47%, H → L + 1 49%
	$S_0 \rightarrow T_1$	H → L + 3 60%, H → L + 1 25%, H → L 7%
10	$S_0 \rightarrow S_1$	H → L 95%
	$S_0 \rightarrow T_1$	H → L 66%, H → L + 1 13%, H → L + 2 7%
11	$S_0 \rightarrow S_1$	H → L 87%, H → L + 1 6%, H → L + 2 3%
	$S_0 \rightarrow T_1$	H → L 74%, H → L + 2 8%
12	$S_0 \rightarrow S_1$	H → L 97%
	$S_0 \rightarrow T_1$	H → L + 3 49%, H → L + 2 20%, H → L + 1 20%

TABLE S8. Comparison of vertical excitation energies (in eV) calculated with the Tamm-Dancoff approximation (TDA) and full TDDFT for selected functionals. $\Delta E^{S_0 \rightarrow S_1}$ and $\Delta E^{S_0 \rightarrow T_1}$ denote singlet-singlet and singlet-triplet excitation energies, respectively.

Molecule	State	B3LYP		B3LYP/D3BJ		B3PW91		TPSSh	
		TDA	Full	TDA	Full	TDA	Full	TDA	Full
**1**	S_1	3.431	3.337	3.386	3.300	3.483	3.387	3.303	3.209
	T_1	2.768	2.697	2.763	2.687	2.799	2.728	2.662	2.608
**2**	S_1	4.297	4.257	4.279	4.240	4.314	4.275	4.134	4.109
	T_1	3.595	3.365	3.602	3.367	3.587	3.318	3.536	3.286
**3**	S_1	3.680	3.659	3.675	3.654	3.663	3.641	3.583	3.587
	T_1	3.035	2.871	3.026	2.864	3.029	2.846	2.943	2.806
**4**	S_1	3.379	3.337	3.336	3.300	3.379	3.336	3.255	3.209
	T_1	2.875	2.802	2.867	2.803	2.854	2.774	2.756	2.683
**5**	S_1	3.829	3.740	3.806	3.722	3.839	3.747	3.686	3.878
	T_1	2.811	2.575	2.815	2.580	2.787	2.523	2.733	2.482
**6**	S_1	4.066	4.053	4.068	4.054	4.089	4.074	3.933	3.920
	T_1	3.750	3.498	3.747	3.499	3.726	3.443	3.642	3.398
**7**	S_1	4.361	4.297	4.342	4.278	4.373	4.305	4.235	4.175
	T_1	3.577	3.366	3.561	3.353	3.547	3.308	3.474	3.249
**8**	S_1	4.144	4.134	4.148	4.138	4.156	4.144	4.026	4.011
	T_1	3.590	3.366	3.592	3.370	3.577	3.323	3.514	3.277
**9**	S_1	4.041	4.029	4.044	4.031	4.056	4.044	3.887	3.876
	T_1	3.616	3.370	3.616	3.371	3.597	3.322	3.548	3.284
**10**	S_1	4.143	4.108	4.137	4.092	4.168	4.129	4.031	3.988
	T_1	3.592	3.386	3.531	3.332	3.527	3.284	3.432	3.214
**11**	S_1	4.175	4.136	4.152	4.111	4.190	4.146	4.071	4.029
	T_1	3.550	3.336	3.525	3.318	3.505	3.267	3.413	3.190
**12**	S_1	4.001	3.995	4.008	4.002	4.003	3.997	3.831	3.827
	T_1	3.621	3.374	3.622	3.376	3.603	3.326	3.564	3.297

TABLE S9. HOMO and LUMO energies (in eV) for molecules 1-12 calculated with different density functionals in combination with def2-TZVPP.

Molecule	Properties	BP86	BLYP	PBE	B3LYP	B1LYP	PBE0	B3PW91	TPSS	TPSSh
1	HOMO	-5.9708	-5.7249	-5.8688	-6.5705	-6.6948	-6.8367	-6.6853	-5.9080	-6.2894
	LUMO	-3.5429	-3.3525	-3.4275	-2.6555	-2.3907	-2.4789	-2.7205	-3.3357	-2.9636
2	HOMO	-5.0160	-4.7768	-4.9155	-5.6284	-5.7491	-5.9147	-5.7502	-4.9414	-5.3269
	LUMO	-1.5558	-1.3109	-1.4662	-0.6684	-0.4221	-0.5734	-0.7606	-1.2903	-0.9482
3	HOMO	-5.9642	-5.7156	-5.8614	-6.5468	-6.6687	-6.8109	-6.6638	-5.8995	-6.2728
	LUMO	-3.0496	-2.8192	-2.9579	-2.1809	-1.9295	-2.0747	-2.2818	-2.8347	-2.4941
4	HOMO	-5.8120	-5.5801	-5.7036	-6.4581	-6.5887	-6.7132	-6.5631	-5.7637	-6.1632
	LUMO	-3.2748	-3.0359	-3.1823	-2.3620	-2.1027	-2.2534	-2.4632	-3.0455	-2.6849
5	HOMO	-5.0554	-4.8149	-4.9543	-5.6113	-5.7230	-5.8629	-5.7259	-4.9783	-5.3376
	LUMO	-2.1981	-1.9559	-2.1039	-1.3711	-1.1355	-1.2908	-1.4826	-1.9738	-1.6606
6	HOMO	-5.2842	-5.0540	-5.1822	-5.9045	-6.0319	-6.1732	-6.0171	-5.2084	-5.6091
	LUMO	-1.9197	-1.6797	-1.8321	-1.0183	-0.7612	-0.9070	-1.1097	-1.6549	-1.3091
7	HOMO	-5.1083	-4.8695	-5.0104	-5.7252	-5.8575	-5.9885	-5.8348	-5.0247	-5.4142
	LUMO	-1.6458	-1.3997	-1.5640	-0.7570	-0.5087	-0.6740	-0.8672	-1.3981	-1.0558
8	HOMO	-5.6072	-5.3758	-5.5073	-6.2397	-6.3712	-6.5083	-6.3482	-5.5400	-5.9343
	LUMO	-2.1511	-1.9117	-2.0698	-1.2213	-0.9626	-1.1215	-1.3286	-1.9036	-1.5432
9	HOMO	-5.3731	-5.1489	-5.2695	-5.9894	-6.1170	-6.2449	-6.0961	-5.3142	-5.6991
	LUMO	-2.0682	-1.8299	-1.9817	-1.1592	-0.9042	-1.0498	-1.2502	-1.8114	-1.4525
10	HOMO	-5.1540	-4.9407	-5.0526	-5.7944	-5.9340	-6.0184	-5.8848	-5.0854	-5.4688
	LUMO	-1.8057	-1.5700	-1.7294	-0.9170	-0.6534	-0.8120	-1.0036	-1.5537	-1.2009
11	HOMO	-4.9887	-4.7627	-4.8902	-5.5962	-5.7221	-5.8454	-5.6977	-4.9133	-5.2908
	LUMO	-1.6079	-1.3516	-1.5319	-0.7129	-0.4589	-0.6306	-0.8225	-1.3582	-1.0123
12	HOMO	-5.2193	-4.9800	-5.1193	-5.8355	-5.9636	-6.1032	-5.9473	-5.1608	-5.5495
	LUMO	-1.9175	-1.6740	-1.8330	-0.9876	-0.7297	-0.8922	-1.0987	-1.6707	-1.3086

TABLE S10. Energy gap (in eV) calculated from HOMO-LUMO energies for molecules 1-12 with different density functionals in combination with def2-TZVPP.

Molecule	BP86	BLYP	PBE	B3LYP	B1LYP	PBE0	B3PW91	TPSS	TPSSh
1	2.4279	2.3724	2.4413	3.9150	4.3041	4.3578	3.9648	2.5723	3.3258
2	3.4602	3.4659	3.4493	4.9600	5.3270	5.3413	4.9896	3.6511	4.3787
3	2.9146	2.8964	2.9035	4.3659	4.7392	4.7362	4.3820	3.0648	3.7787
4	2.5372	2.5442	2.5213	4.0961	4.4860	4.4598	4.0999	2.7182	3.4783
5	2.8573	2.8590	2.8504	4.2402	4.5875	4.5721	4.2433	3.0045	3.6770
6	3.3645	3.3743	3.3501	4.8862	5.2707	5.2662	4.9074	3.5535	4.3000
7	3.4625	3.4698	3.4464	4.9682	5.3488	5.3145	4.9676	3.6266	4.3584
8	3.4561	3.4641	3.4375	5.0184	5.4086	5.3868	5.0196	3.6364	4.3911
9	3.3049	3.3190	3.2878	4.8302	5.2128	5.1951	4.8459	3.5028	4.2466
10	3.3483	3.3707	3.3232	4.8774	5.2806	5.2064	4.8812	3.5317	4.2679
11	3.3808	3.4111	3.3583	4.8833	5.2632	5.2148	4.8752	3.5551	4.2785
12	3.3018	3.3060	3.2863	4.8479	5.2339	5.2110	4.8486	3.4901	4.2409

All cartesian coordinates, in Å, of the optimized DS12 set geometries shown in this file were calculated using the B3LYP/def2-TZVPP level.

Molecule 1

C	-8.97395756703420	-0.99270232603618	0.02310966028367
C	-8.35232800655581	-0.76299901371406	-1.21224447963034
C	-8.72863807726856	-1.56675049507605	-2.29164268840123
C	-9.68024445508973	-2.56783960886690	-2.16204655372514
C	-10.27203788530591	-2.77199978410325	-0.92258759754566
C	-9.92646633209507	-1.99387150985947	0.17383149046752
S	-7.26893303822785	0.62097245729004	-1.52795491903610
C	-5.71263520584527	0.06377903471898	-0.88142924995041
C	-4.59128871717301	0.91293595017023	-0.79860499907302
C	-3.37170893192810	0.47886812883008	-0.28246344973516
C	-3.22740420461549	-0.82069190691813	0.15975543159870
C	-4.31834291349304	-1.68327172862380	0.08095615083155
C	-5.53086881482302	-1.24989886203796	-0.42612723756880
N	-4.65066530562619	2.29946313469359	-1.25971207511257
O	-5.67149055692143	2.65538465832539	-1.84021775023429
O	-3.69289170644808	3.02840411660758	-1.04919320039160
H	-8.26362657476674	-1.39276247758843	-3.25234481257338
H	-9.95971509959087	-3.17833987372498	-3.00804327050010
Cl	-11.47148736355877	-4.01854065509425	-0.73123183889417
H	-10.39451437139925	-2.15663877205149	1.13267284039822
H	-2.55683368385798	1.18503569270568	-0.24436495325648
H	-4.22623647855819	-2.70782387535609	0.41684052697443
H	-6.35557172638321	-1.94357888826715	-0.47535499823886
Cl	-8.57744525318222	-0.04052962985941	1.42136751547843
H	-2.28076773025175	-1.15910376616398	0.55643045783481

Molecule 2

C	-11.30395744646753	3.56009134276647	1.63726390664513
C	-10.17838083089196	2.80088092462594	1.90882499118669
C	-9.35379558388633	2.32247718173488	0.86720438523584
C	-9.73319847777162	2.64227165960293	-0.45472008206877
C	-10.86082739889191	3.40402508222006	-0.70988693325928
C	-11.66939133024331	3.87216419633579	0.32703873095291
S	-13.07540928828675	4.90377978581183	-0.00607433005014
N	-8.22463307992347	1.58142992617926	1.12967581626055
C	-14.43168197819123	3.76753083563521	-0.20873369716441
C	-14.31748399328852	2.38105845910893	-0.11848738436293
C	-15.43202526569116	1.56825910236770	-0.29308381046480
C	-16.66738879678208	2.13979189736951	-0.55880824820765
C	-16.79969429348255	3.52025355880700	-0.65181371254022
C	-15.68535714433899	4.32693479029918	-0.47695893106918
H	-11.91263915140857	3.91705262696095	2.45746634481503
H	-9.93937145568202	2.58366674236017	2.93841474433968
H	-9.14388293098803	2.29865036740476	-1.29077411619112
H	-11.12097248348509	3.63849089855107	-1.73362039084790
H	-13.35917926129193	1.92718322630698	0.08818630473448
H	-15.33742013824866	0.49377629096882	-0.22182629982698
Cl	-18.06907819170973	1.11773817671952	-0.77814670746809
H	-17.76540169716156	3.95935626707251	-0.85872601848986
C	-7.45083844230852	1.02325381127280	0.03760841016039
C	-7.91300245398030	1.18668428728378	2.48973233653070
H	-8.02781589448082	0.30847927678692	-0.56086993782736
H	-6.98157776142283	0.62710785617434	2.49401815712250
H	-6.58707771339506	0.50302477001983	0.44252926065006
H	-8.69222717364857	0.55295811384838	2.92878932279459
H	-7.08388456941065	1.80497275869749	-0.63265821024027
H	-7.78159095816521	2.05665864650588	3.13885796045596

H -15.79241381507384 5.40189914020089 -0.54992086180556

Molecule 3

C	-8.97214941300500	-1.08080618873399	0.10330266734902
C	-8.36999855854187	-0.74378391439133	-1.11769800062502
C	-8.75346339944329	-1.45444066048027	-2.25794237461814
C	-9.69604587316308	-2.47029351447054	-2.20131886683217
C	-10.26643212774494	-2.78695308946375	-0.97530037759010
C	-9.91191566671855	-2.10208380009879	0.17913457503703
S	-7.27230783354147	0.64169297462897	-1.31138567913770
C	-5.71507508784146	0.04760899545489	-0.71503725309536
C	-4.64854869007467	0.95246065376342	-0.62580913769867
C	-3.40400397125478	0.54783987230914	-0.17562030505827
C	-3.18853714974325	-0.77759335589348	0.19870049754993
C	-4.24175378831158	-1.69171060222309	0.10710189198738
C	-5.48152005058458	-1.28499703947533	-0.34366195126909
H	-8.30064587858160	-1.19673398959981	-3.20527181171694
H	-9.98230380278148	-3.00876938646673	-3.09266050837746
Cl	-11.44884920040766	-4.05861170419886	-0.87411802816327
H	-10.36377222177295	-2.35253281734811	1.12676112150806
H	-2.59681586069692	1.26606049918931	-0.11301113436150
H	-4.06854125489450	-2.72017516385132	0.39321943425064
H	-6.28546898147963	-2.00267567516602	-0.41497245197539
Cl	-8.56929799533476	-0.24256841904652	1.56890552409151
Br	-4.87968178049034	2.77943834254804	-1.13481015329114
C	-1.85568586358987	-1.18725572862378	0.68132385373128
H	-1.10320850111445	-0.37283308685190	0.70212490962903
O	-1.56277704888725	-2.30528320151014	1.03534355867634

Molecule 4

C	-8.85403402675645	-0.95769807059558	-0.00392519626096
C	-8.31400496048722	-0.76100694460916	-1.28393570572170
C	-8.79503634080274	-1.55560137579091	-2.32802008834907
C	-9.77363909551027	-2.51704096725744	-2.12283253057137
C	-10.27671668915923	-2.69802910538948	-0.84149998285673
C	-9.82355470861802	-1.92757422820935	0.22105672953126
S	-7.16003082822627	0.52949853153953	-1.68026513400735
C	-5.64302488316000	0.01083819695837	-0.89508710458435
C	-4.66025240461234	0.96720556137969	-0.57858221415526
C	-3.43709713560765	0.55408298431329	-0.03724557354540
C	-3.20066002997066	-0.78257527848951	0.20654060422752
C	-4.16968465348584	-1.73563015299810	-0.09560746512701
C	-5.37536674458961	-1.33745469881793	-0.65002976689561
H	-8.38716418645811	-1.41028376909640	-3.31871147182121
H	-10.13510149314207	-3.12095818254824	-2.94202378922868
Cl	-11.49629685264906	-3.90508509172894	-0.55492604520164
H	-10.22492002940345	-2.07009828800857	1.21285111655756
H	-2.70161874901052	1.31206915205602	0.19166044376562
H	-3.98211735414968	-2.78332786125601	0.09179152319965
H	-6.11136613519090	-2.08892982107067	-0.89678613423515
Cl	-8.34075354978976	-0.00123546202404	1.35075404170752
C	-4.86807472240490	2.42468779694907	-0.76949083894302
Br	-1.54005146898281	-1.33776663708347	0.96527150050907
O	-4.00352072954076	3.25500182553874	-0.61163999409188
H	-5.88911222829153	2.72901188623910	-1.05781692390280

Molecule 5

C	-0.71532378101657	0.32746829351403	-0.65149893121719
C	0.42679465248407	-0.39989856398706	-0.94730122704620
C	1.06615035879403	-1.15540351170260	0.04286074171230
C	0.52307506095514	-1.15972797465259	1.33479887516545
C	-0.62195472957168	-0.43535642574435	1.61023229766578
C	-1.27701252087339	0.32315736828118	0.63359046364767
C	-5.33020082206228	0.99168751984291	0.69015596184640
C	-4.08012590979643	0.42249391910303	0.54620759159118
C	-3.98595611330890	-0.86682581887501	-0.03161024021355
C	-5.10290287221595	-1.53464324919639	-0.44721210763529
C	-6.39120024211970	-0.96701160019730	-0.31163579625455
C	-6.51772828852229	0.32981025393338	0.27591153674851
C	-7.81436760542726	0.88517302231465	0.41028742292270
C	-7.55613061546424	-1.64646550455865	-0.73949174057894
C	-8.79676400382001	-1.08131133644683	-0.59779174041511
C	-8.92174229691854	0.19654940951882	-0.01562712665685
Cl	-5.45317940952729	2.57996944522012	1.41274516944578
S	-2.65727377149987	1.33087037464681	1.10819244139084
Cl	-1.44094601895869	1.24184246346280	-1.94307372727193
N	2.23604116347857	-1.83559168059582	-0.24033355450308
H	2.42604818076633	-2.04044819624095	-1.20653470572505
H	2.49565101638055	-2.57686669416863	0.38791306783526
H	0.82648212032485	-0.37040919004100	-1.95180800668190
H	1.00371988235441	-1.72943345397398	2.12011891053283
H	-1.03333265257766	-0.44631774853965	2.61036817141231
H	-3.01267839279207	-1.32140754260155	-0.14153848890265
H	-5.00812157880404	-2.51830477557147	-0.88994955465173
H	-7.92287031993472	1.86331475587568	0.85420588768255
H	-7.44707010128153	-2.62784266695835	-1.18457414491926
H	-9.67951803147411	-1.61132642319146	-0.93020455545596

H	-9.90256235757058	0.64005553153028	0.09639710852968

Molecule 6

C	-10.90449427464184	4.01032454593505	-0.53716329002470
C	-9.83145668067854	4.18568388780471	0.32989880250335
C	-9.39854768212368	3.10222065061817	1.07290789884961
C	-9.99876948014267	1.85647892663572	0.98372897327890
C	-11.06050291938484	1.69161577004155	0.10428572732615
C	-11.52371696864129	2.76578079430622	-0.65991312987906
S	-12.80184039430811	2.53710794291071	-1.87944627859178
F	-8.35215144369030	3.26140559971083	1.90823814789039
C	-14.32097054340415	2.46517661562951	-0.95006624913374
C	-15.45577535769598	2.06433440725514	-1.66227170419421
C	-16.69199957354873	2.01025932696813	-1.03438184832939
C	-16.81415361193250	2.34472451966578	0.31118506418535
C	-15.68488808369073	2.73810596564017	1.01905090252429
C	-14.44216856219800	2.80435288668522	0.39659019518162
H	-11.26115863284042	4.84256143809160	-1.12787279719010
H	-9.33624255141705	5.14110922927055	0.43196663690881
H	-9.62653053406324	1.03640023121328	1.58173441657072
H	-11.52948052568804	0.72266418369886	0.00309324924404
H	-15.36733022441076	1.79284615455223	-2.70672904571581
H	-17.56150250281804	1.69843457998920	-1.59860444073705
H	-17.77760484827653	2.29724059759425	0.80060300527545
H	-15.76601824452060	3.00200525062637	2.06579321326632
H	-13.57569635988307	3.11976649515657	0.95997255079086

Molecule 7

C	-10.42693047215290	3.79705845305995	-0.69597609916617
C	-9.29285407927984	3.54297845765410	0.08308257610688
C	-9.19961211776320	2.35717218620675	0.80840039066759
C	-10.24020750469744	1.43083043563163	0.73213150638452
C	-11.35592286485350	1.66149045649592	-0.05348782439562
C	-11.44935036538513	2.86067123151775	-0.76948752553257
S	-12.80337483978925	3.19625843911108	-1.87984034665969
C	-14.25577577117493	2.79650872439782	-0.92811267490649
C	-15.30726656769349	2.15301230572459	-1.58273507793853
C	-16.49823284284777	1.89903438324073	-0.91068105875827
C	-16.64271379801099	2.26432868201227	0.42234098786856
C	-15.59101210757032	2.89905914850723	1.07808373360543
C	-14.40738372256309	3.17605133486765	0.40746861495065
H	-10.49259157534789	4.73459446894547	-1.23045982109512
H	-10.16356152892831	0.50704728605739	1.29121425049003
H	-12.14423224308309	0.92549382222017	-0.11721843917997
H	-15.18704269421057	1.84679014487042	-2.61350999014320
H	-17.30763042854229	1.40149865144388	-1.42912744497029
H	-17.56560007869875	2.05674742503820	0.94744639390307
H	-15.69656995451523	3.19194835347146	2.11484398196374
H	-13.60172703297323	3.68576013445142	0.91750638717872
O	-8.34747072322437	4.52200149644720	0.07770275191497
C	-7.18215880518592	4.34644249525676	0.86693678339006
H	-8.33917618952569	2.14430594020228	1.42423280526947
H	-6.58155987008070	5.23970878698697	0.71643179255445
H	-6.61152738330472	3.46976269944062	0.54886272129133
H	-7.42881443859589	4.25144405673999	1.92795062520644

Molecule 8

C	-10.41682015753883	3.88132718629451	-1.10356075730632
C	-9.21551991903338	3.38014238071759	-0.62786509030075
C	-9.20804587522892	2.40686048118161	0.36766193572067
C	-10.41634751211095	1.94329597365120	0.88106702593440
C	-11.62103713691880	2.44527809022853	0.41172641087421
C	-11.63235146366006	3.41957847543630	-0.58821384992306
S	-13.10424684788035	4.16081268632222	-1.25139014522493
C	-14.43307291831564	3.16993549156456	-0.59158021761755
C	-14.78067892881498	1.96230927078824	-1.19971481251283
C	-15.85194876118967	1.22300735658508	-0.71355231699370
C	-16.58826860595458	1.68960373530758	0.37150808351660
C	-16.25113685093212	2.89775676146604	0.97130799863088
C	-15.17432453710422	3.63779878444333	0.49337405172437
H	-10.41176176934424	4.63345055656083	-1.88177292535644
H	-10.42003549464883	1.19133331005616	1.65855351085790
H	-12.54794176571089	2.07928349422307	0.82781813137143
H	-14.21302241182008	1.60936461642381	-2.04989066542560
H	-16.11566885098155	0.28629269914918	-1.18678993335881
H	-17.42531220689462	1.11462124931869	0.74515812780720
H	-16.82321735232893	3.26614585643856	1.81263823769098
H	-14.90525230602601	4.57702540859276	0.95702120124090
C	-7.91863426652259	1.81760517417446	0.86254670776337
F	-7.69000943992136	0.58818524775999	0.33763196216667
F	-7.91401412843134	1.66072264224227	2.20536610509471
F	-6.84994572448973	2.57654410718258	0.54729324997604
H	-8.28358476819629	3.74681896389062	-1.03374202635033

Molecule 9

C	-10.92995987384269	4.03783188064371	-0.47839494767070
C	-9.88416196727467	4.23397225498450	0.41670384986448
C	-9.42948581950990	3.15185464847569	1.14904247898144
C	-9.98223671332242	1.88748770069555	1.02254629506676
C	-11.01812367340862	1.70254439611760	0.11668019864237
C	-11.50196316528561	2.77480601321247	-0.63723168235827
S	-12.74700962344704	2.52286353969293	-1.88612353828334
C	-14.28348593392572	2.44217453619993	-0.99045318233022
C	-15.40558073682197	2.04596646897966	-1.72475299660668
C	-16.65536328006925	1.98035984972051	-1.12794914898627
C	-16.78987510771227	2.30433214559714	0.21697290919236
C	-15.68560009302652	2.69456244699801	0.96051897239644
C	-14.43522896275481	2.76715663607607	0.35627528894358
H	-11.30138025794958	4.86807863045180	-1.06279157035511
H	-9.59383230575757	1.06959755281766	1.61313525944759
H	-11.44975814065634	0.71972900283687	-0.01301231527894
H	-15.30282373328493	1.78422279303433	-2.77021113851451
H	-17.51887737617028	1.67363693896769	-1.70085726314709
H	-15.79889973961575	2.94538723389847	2.00573395153706
H	-13.58377762619956	3.07911126741681	0.94361745377450
H	-9.42535019571514	5.20396271022861	0.54724680182730
F	-8.40917537488536	3.33119113573896	2.01087408701488
Cl	-18.36105029936314	2.21707021721485	0.97483023684238

Molecule 10

C	-10.55909451117250	4.22041739609842	-0.47583909762715
C	-9.45720479427700	3.98172653308428	0.33688847641449
C	-9.41139694203191	2.80791401412704	1.06697881778870
C	-10.42831660923323	1.87064697884956	1.00867123157029
C	-11.51639350973666	2.10844195641683	0.17818167493226
C	-11.59100727710348	3.28386927424904	-0.57120137453280
S	-12.89655968454435	3.62291839919551	-1.73714047380611
C	-14.33076510634642	2.82834677150293	-1.03743527006836
C	-14.98416313602632	1.83787709987811	-1.77158513539983
C	-16.15802098083260	1.26958336791945	-1.29282006464519
C	-16.70210985902387	1.65752108984506	-0.06735333199086
C	-16.03365798268997	2.64343747366171	0.66181021038584
C	-14.87083918487770	3.23283805567365	0.18333053847322
H	-10.61783488214939	5.14267776890979	-1.03857383978231
H	-10.35936669063650	0.96626530930642	1.59726318192403
H	-12.30810640853661	1.37517398583703	0.11701791264084
H	-14.56933411569736	1.51641528807472	-2.71752425739872
H	-16.65372148743176	0.50613841228453	-1.88074835693480
H	-16.43819698917566	2.96786522542923	1.61296363467666
H	-14.38396612942599	4.01110727446908	0.75543235441321
H	-8.65129969842853	4.69798979459168	0.41589812887836
F	-8.34410552055069	2.56931859501987	1.86177105535307
C	-17.97360000201333	1.03253709270083	0.44403082930172
H	-18.26375123803885	1.45276230852184	1.40624826283025
H	-17.86124898503334	-0.04663414770709	0.56968814794922
H	-18.79903827498532	1.19014468206037	-0.25355325534605

Molecule 11

C	-10.53558873028597	4.04245082406593	-0.69173934225325
C	-9.40409426271891	3.68512625421204	0.02909120019784
C	-9.30728364228479	2.44475375960884	0.66254072936933
C	-10.38747809795782	1.57018227601430	0.53972964141363
C	-11.51750683844534	1.90648039685399	-0.19562991936028
C	-11.60420741064955	3.15194654204459	-0.81568492800384
S	-12.97728378394422	3.64849685425330	-1.83809617763278
C	-14.38539738544743	2.84943779442642	-1.09436026663961
C	-15.17777613687153	2.00973559445569	-1.87287241698034
C	-16.33088585627783	1.43969940010877	-1.34065456227058
C	-16.71205503152843	1.67677930983945	-0.02221014130188
C	-15.90302999705614	2.51623771189814	0.75194067822479
C	-14.76464448358742	3.10587033807163	0.22600829651390
H	-10.59559893152855	5.01989255621107	-1.15288114066347
H	-10.34492838205340	0.60044468522526	1.02147643276316
H	-12.33175795557015	1.20032973809623	-0.28106151680470
H	-14.88850696903801	1.79816774319898	-2.89359107570724
H	-16.93454150833214	0.79002313340881	-1.96275968717529
H	-16.17702363527336	2.72113256250517	1.78025590955949
H	-14.16428383168910	3.76560346849598	0.83781304809130
H	-8.58810708813223	4.39345017167325	0.11274852640791
C	-17.96190347927753	1.06616753219850	0.55470339214428
H	-17.77079288119152	0.61617004328196	1.53053556292541
H	-18.36609209085382	0.29450635205438	-0.09980970723868
H	-18.73937463604714	1.82152342781940	0.69537892593016
C	-8.07215006695954	2.06148708232676	1.43555236389477
H	-8.26163145388515	1.21150793762113	2.09092153063612
H	-7.71405657132735	2.88867695402696	2.05066915526455
H	-7.25611886178449	1.78311955600290	0.76318548869529

Molecule 12

C	-10.99608592739709	3.70813553938605	-0.55941001679812
C	-9.88041597385617	3.69136323005007	0.26846269212710
C	-9.29953932654827	2.48871212452107	0.67457317730759
C	-9.87535446797268	1.29723618313372	0.22786020878936
C	-10.99038174515526	1.30338985554966	-0.59983034093433
C	-11.56397590301169	2.51183010205938	-0.99635276336496
S	-12.94920913904248	2.52812783683322	-2.11717820176709
C	-14.37745879093193	2.50090158835675	-1.05522524574641
C	-15.62431136128346	2.50174768950263	-1.68938659118899
C	-16.79635394713591	2.48387392084077	-0.94868125580036
C	-16.72960162956268	2.46450871372290	0.43955906526885
C	-15.50148706486196	2.46246362215268	1.08383556160111
C	-14.32829496318694	2.48074839798630	0.33757367205772
H	-11.43090918817781	4.64911813299991	-0.86818851629374
H	-9.44484953376950	0.34997171614289	0.52952643005238
H	-11.42087625106653	0.37150857942244	-0.94062513134464
H	-15.68095812997293	2.51644914354720	-2.77048851331511
H	-17.75618114156658	2.48499311276398	-1.44548606525107
H	-15.45739388788940	2.44703609288224	2.16372579623883
H	-13.37796858678686	2.47940566112047	0.85107015278388
H	-9.45421876269809	4.62971444755763	0.60203359229543
C	-8.06984291254261	2.47503593756064	1.54351606676459
H	-8.06827200208253	1.61856067546181	2.21830616764157
H	-7.99534385141653	3.38193887313571	2.14338984386801
H	-7.16419512496460	2.41029609683048	0.93442031964289
Cl	-18.20342038711861	2.44353272647919	1.37839989536552
